# Integrative characterisation of secreted factors involved in intercellular communication between prostate epithelial or cancer cells and fibroblasts

**DOI:** 10.1002/1878-0261.13376

**Published:** 2023-01-27

**Authors:** Yunjian Wu, Kimberley C. Clark, Birunthi Niranjan, Anderly C. Chüeh, Lisa G. Horvath, Renea A. Taylor, Roger J. Daly

**Affiliations:** ^1^ Cancer Program, Biomedicine Discovery Institute Monash University Clayton Victoria Australia; ^2^ Department of Biochemistry and Molecular Biology Monash University Clayton Victoria Australia; ^3^ Department of Anatomy and Developmental Biology Monash University Clayton Victoria Australia; ^4^ Garvan Institute of Medical Research Darlinghurst New South Wales Australia; ^5^ University of Sydney New South Wales Australia; ^6^ Chris O'Brien Lifehouse Sydney New South Wales Australia; ^7^ Department of Physiology Monash University Clayton Victoria Australia; ^8^ Cancer Research Division, Peter MacCallum Cancer Centre The University of Melbourne Victoria Australia

**Keywords:** cell signalling, chemokine, co‐culture, cytokine, follistatin, tumour microenvironment

## Abstract

Reciprocal interactions between prostate cancer cells and carcinoma‐associated fibroblasts (CAFs) mediate cancer development and progression; however, our understanding of the signalling pathways mediating these cellular interactions remains incomplete. To address this, we defined secretome changes upon co‐culture of prostate epithelial or cancer cells with fibroblasts that mimic bi‐directional communication in tumours. Using antibody arrays, we profiled conditioned media from mono‐ and co‐cultures of prostate fibroblasts, epithelial and cancer cells, identifying secreted proteins that are upregulated in co‐culture compared to mono‐culture. Six of these (CXCL10, CXCL16, CXCL6, FST, PDGFAA, IL‐17B) were functionally screened by siRNA knockdown in prostate cancer cell/fibroblast co‐cultures, revealing a key role for follistatin (FST), a secreted glycoprotein that binds and bioneutralises specific members of the TGF‐β superfamily, including activin A. Expression of FST by both cell types was required for the fibroblasts to enhance prostate cancer cell proliferation and migration, whereas FST knockdown in co‐culture grafts decreased tumour growth in mouse xenografts. This study highlights the complexity of prostate cancer cell–fibroblast communication, demonstrates that co‐culture secretomes cannot be predicted from individual cultures, and identifies FST as a tumour‐microenvironment‐derived secreted factor that represents a candidate therapeutic target.

## Graphical abstract

This study explored intercellular communication between prostate fibroblasts and prostate cancer cells by integrating secretome characterisation using antibody arrays with functional screens. Follistatin, which bioneutralises activin A, was validated as a key factor required for fibroblasts to promote prostate cancer cell proliferation and migration *in vitro*, and for xenograft growth from co‐culture grafts, and therefore represents a potential therapeutic target.

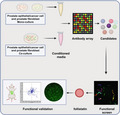

**Abbreviations**
CAFs: carcinoma‐associated fibroblasts
CRISPRi: clustered regularly interspaced short palindromic repeats interference
ECM: extracellular matrix
EMT: epithelial‐mesenchymal transition
FAC: fluorescence‐activated cell
FBS: fetal bovine serum
FST: follistatin
GDF15: growth differentiation factor 15
GEPIA: gene expression profiling interactive analysis
hrFST: human recombinant follistatin
NPFs: non‐malignant prostate fibroblasts
SD: standard deviation
STR: short tandem repeat
TCGA: the cancer genome atlas
TGFα: transforming growth factor alpha
TME: tumour microenvironment

## 1 Introduction

Prostate cancer affects millions of men worldwide, and is the second most common cancer in men, causing > 350 000 deaths per annum globally [1, 2]. Understanding the pathophysiological mechanisms of prostate cancer progression is the key to developing novel therapeutic strategies. Strong evidence has emerged implicating the tumour microenvironment (TME) in cancer development and progression [3, 4], and intercellular communication within the TME is a key regulator of the malignant behaviour of prostate cancer cells. Unique characteristics of the prostate TME, comprised of extracellular matrix (ECM) and diverse stromal cell types, distinguish it from corresponding normal tissue. Carcinoma‐associated fibroblasts (CAFs) are one of the most abundant stromal components in the TME with numerous studies demonstrating their prominent roles in prostate cancer pathogenesis and progression [5, 6, 7, 8], however the specific mechanisms of intercellular dialogue between CAFs and prostate epithelial, or prostate cancer cells, require further characterisation. These complex mechanisms can involve cell–cell contact, cell‐matrix interplay or paracrine communication mediated by soluble factors [9]. Deciphering the mechanisms underpinning tumour stroma‐cancer cell interactions during prostate cancer progression will enable the identification of novel biomarkers and/or treatment strategies.

The emergence of single‐cell technologies has enabled the identification of different CAF subtypes within the TME [10]. Two important CAF subpopulations are termed “myCAFs” and “iCAFs”. The myCAFs exhibit a matrix‐producing contractile (myofibroblast) phenotype, while the iCAFs generate an immunomodulating secretome. CAF‐derived soluble factors mediate autocrine and paracrine signalling, stimulate cancer cell proliferation, enhance epithelial‐mesenchymal transition (EMT) to facilitate metastasis, and increase neovascularisation [5, 11]. Prostate cancer cells that come into contact with CAFs activate the same pro‐inflammatory gene signature and become invasive through EMT [12]. For example, increased secretion of CCL2 and IL‐8 from CAFs in advanced prostate cancer promotes cancer cell migration [13]. Meanwhile, IL‐8 secreted by PTEN‐deficient prostate cancer cells can also augment CAF‐derived CCL2 and CXCL12, leading to increased proliferation and migration of the cancer cells [14]. Thus, the interaction between stromal and tumour cells *results* in an altered secretome, that induces pro‐tumourigenic activities.

To date, various approaches have been employed to investigate the expression of secreted factors from cancer cells and CAFs, including Luminex assays, antibody (cytokine/chemokine) arrays and mass spectrometry. Of note, studies using these methods usually only focus on the secreted factors from one cell type and one culture condition (i.e. utilising mono‐cultures) [15, 16]. However, the secretome is likely to be altered in a co‐culture environment, where reciprocal intercellular signalling is active. In order to address this, we have systematically profiled the secretomes of prostate epithelial and prostate cancer cells, and patient‐derived normal prostatic fibroblasts (NPFs) and CAFs, and compared the secretomes from individual cell types with corresponding co‐cultures. This approach revealed marked changes in the co‐culture secretomes, with markedly elevated expression of several factors compared to mono‐cultures. Follistatin (FST), which can bind and bioneutralise specific members of the TGF‐β superfamily, including activin A [17, 18], was identified as one such factor. Previous studies identified increased serum level of FST in prostate cancer patients [19], compared to that in healthy male and benign prostate hyperplasia patients. In addition, an increased serum level of FST significantly correlated with the presence of bone metastases and higher prostate specific antigen levels [19]. These findings suggested a role for FST in prostate cancer development and progression, but detailed insights into FST regulation and function in prostate cancer have been lacking. In this study, we identify FST as a key regulator of prostate epithelial/cancer cell‐fibroblast intercellular communication, highlighting it as a potential target for therapeutic intervention.

## 2 Materials and methods

### 2.1 Human samples and study approval

Human prostate tissue samples were collected from patients undergoing radical prostatectomy surgery between November 2012 and April 2016, with approval from the following human ethics committees: Cabrini Institute (No. 03‐14‐04‐08), Epworth HealthCare (34306 and 53611) and Monash University (No. 2004/145). All methods involving human participants were performed in accordance with the ethical standards of the institutional committee and with the 1964 Helsinki declaration and its later amendments and all experiments were undertaken with the understanding and written informed consent of each subject. Prostate cancer patient mRNA data was obtained through the Gene Expression Profiling Interactive Analysis (GEPIA) [20] and TCGA.

### 2.2 Human samples and cell culture

The human prostate cancer cell lines PC3 and 22RV1, human prostate epithelium cell line BPH‐1, prostate stroma fibroblast line WPMY‐1 and HEK293T cells were obtained from ATCC (*In Vitro* Technologies, Melbourne, Vic., Australia). PC3, BPH‐1 and WPMY‐1 cell lines were cultured in RPMI 1640 medium (School of Biomedical Sciences, Media and Prep Services, Monash University) supplemented with 5% (v/v) fetal bovine serum (FBS, Invitrogen, Waltham, MA, USA). 22RV1 was cultured in RPMI 1640 medium containing 10% (v/v) FBS. HEK293T cells were cultured in DMEM medium (School of Biomedical Sciences, Media and Prep Services, Monash University) supplemented with 5% (v/v) FBS. Primary fibroblasts (CAFs and NPFs) were isolated from patient specimens as previously described [21] and cultured in fibroblast media (RPMI 1640) supplemented with phenol red, 5% (v/v) heat inactivated HyClone fetal bovine serum (HI‐FBS; GE Healthcare, Chicago, IL, USA), 1 nm testosterone (T1500, Sigma‐Aldrich, St. Louis, MO, USA), 10 ng·mL^−1^ basic fibroblast growth factor (100‐18B; Peprotech, Rochy Hill, NJ, USA, Lonza Pharma & Biotech, Brooklyn, Victoria, Australia) and 100 UI·mL^−1^ penicillin/10 μg·mL^−1^ streptomycin (15140‐122, Gibco™, Thermo Fisher Scientific, Waltham, MA, USA). Cells were maintained at 37 °C in 5% CO_2_, 5% O_2_ atmosphere, with media changes every 2–3 days. Prior to this study the two pairs of patient matched CAFs and NPFs (128R and 332R) were validated by *in vivo* tissue recombination experiments and tumourigenicity of primary CAFs was confirmed as previously described [21, 22]. All patient information can be found in Table S1. All primary patient‐derived fibroblasts were used at early passages (3–8) unless otherwise stated. All cell lines tested negative for Mycoplasma, and cell line authentication was performed via short tandem repeat (STR) profiling by Cell Bank Australia.

### 2.3 Preparation of conditioned media

Cells were seeded in 10 cm culture dishes at 1.6 × 10^6^ cells per dish, and 5 × 10^4^ cells per well for each cell type (5 × 10^4^ epithelial cells and/or 5 × 10^4^ fibroblasts) in a 12 well plate for co‐culture conditioned media (or Transwell conditioned media) in complete medium. Upon reaching 80–90% confluence, cells were washed with PBS and the medium replaced with 8 mL serum‐free medium for 10 cm dishes, and 1 mL serum‐free medium for 12 well plate. After 48 h, the conditioned media were collected, centrifuged and stored at −80 °C until analysis.

### 2.4 Expression of fluorescence markers and luciferase

The expression vector for Luciferase/mCherry (pRV100G ofl T2A Luciferase/mCherry) was kindly provided by Prof. Paul Timpson (Garvan Institute, NSW, Australia). The expression vector pGIPZ for GFP was purchased from Thermo Fisher Scientific. Lentiviral or retroviral transduction of PC3, BPH‐1 and 22RV1 cells for GFP, mCherry and Luciferase expression was performed as previously described [23] by using Lipofectamine™ 3000 reagent (Thermo Fisher Scientific). Positive cells were selected by Fluorescence‐activated cell (FAC) sorting.

### 2.5 siRNA transfection

Cells were transfected for 24 h with siRNA pools targeting CXCL10, CXCL6, CXCL16, FST, PDGFA or IL‐17B (Dharmacon, Lafayette, CO, USA), or ON‐TARGETplus (OTP) as the negative control. Sequences of siRNAs are shown in Table S2. Cells were used for further functional experiments within 48 h after transfection.

### 2.6 CRISPRi‐mediated FST knockdown

PC3 and WPMY‐1 cells were infected with dCas9‐KRAB lentivirus produced in HEK293T cells, which were transiently transfected with dCas9‐KRAB plasmid (89567, Addgene, Watertown, MA, USA) and packaging plasmids (pMD2.G, 12259 and psPAX2, 12260, Addgene) using Lipofectamine™ 3000 reagent. Cells were subjected to Blasticidin (A1113903, Thermo Fisher Scientific) selection (40 μg·mL^−1^ for PC3 cells, 5 μg·mL^−1^ for WPMY‐1 cells) to generate stable dCas9‐KRAB expressing cell clones. Expression of dCas9‐KRAB was validated via western blot using an anti‐Cas9 antibody (SAB4200701, Sigma‐Aldrich). Five pairs of sgRNAs targeting the human FST gene were designed using the Broad Institute's GPP sgRNA designer (https://portals.Broadinstitute.org/gpp/public/analysis‐tools/sgrna‐design) and cloned into the pXPR003 backbone (52963, Addgene) and sequence validation was performed. dCas9‐KRAB cells were then infected with sgFST‐containing pXPR003 (or empty vector pXPR003) lentivirus followed by Puromycin (A1113802, Thermo Fisher Scientific) selection. FST knockdown was validated at the protein level by western blot using an anti‐FST antibody (ab157471, Abcam, Cambridge, UK). All CRISPR guide sequences and primer information are listed in Table S3.

### 2.7 Random cell migration assay

PC3‐GFP cells (1.8 × 10^4^/well) or PC3‐GFP cells + WPMY‐1 cells (1.8 × 10^4^ + 1.8 × 10^4^/well) were seeded in a 12 well plate as mono‐culture or co‐culture. The selected targets (CXCL10, CXCL6, CXCL16, FST, IL‐17B and PDGFA) were knocked down in PC3‐GFP cells and WPMY‐1 cells, individually or in combination, by siRNA (or CRISPRi) before seeding. After 24 h, the medium was replaced with serum‐free medium with 1 μg·mL^−1^ Mitomycin C (Sigma‐Aldrich). Human recombinant CCL11 (Peprotech) (8 ng·mL^−1^) was added in PC3‐GFP mono‐culture as the positive control for migratory promotion and a FAK inhibitor (FAKi, PF‐562271, Sigma‐Aldrich) was added to the co‐cultures as the positive control for migratory inhibition. Human recombinant FST (hrFST) protein (R&D Systems, Minneapolis, MN, USA) at different concentrations (10, 50 and 100 ng·mL^−1^) was used in the rescue experiments. Random cell migration was monitored using a Leica DMi8 Live Cell microscope (Wetzlar, Germany). Three fields per well were chosen under a 10× magnification objective. Time‐lapse movies of the PC3‐GFP cells in each mono‐culture and co‐culture condition were recorded over 24 h with an image acquired every 20 min. The movie files were analysed using the MtrackJ plug‐in of the imagej software (National Institutes of Health, Bethesda, MD, USA). This method was repeated to validate the FST knockdown effect in 22RV1‐mCherry cells and BPH‐1‐GFP cells.

### 2.8 Proliferation assay

PC3‐GFP cells (5000/well) or PC3‐GFP + WPMY‐1 cells (5000 + 5000/well) were seeded in a 96 well plate as mono‐ or co‐cultures for 3–5 days. The number of green fluorescent cells was recorded and analysed by imagej. In the proliferation rescue experiment, PC3 + WPMY‐1 co‐cultures or PC3_FST_KD + WPMY‐1_FST_KD co‐cultures were seeded at day 0, then three different concentrations (10 ng·mL^−1^, 50 ng·mL^−1^ and 100 ng·mL^−1^) of hrFST protein were added into the co‐culture system at day 1, and culture continued for 3 days. The number of GFP positive cells was counted using imagej software.

### 2.9 Antibody array analysis

Cytokine analysis was performed using the Human Cytokine Array Q4000 (RayBiotech, Norcross, GA, USA) which included 200 human inflammation factors, growth factors, chemokines, cytokines and receptors. Then, a customised antibody array which included 24 prioritised proteins from the Q4000 array was analysed. Briefly, the arrays were blocked with the blocking buffer (provided by the kit) for 30 min and incubated with 1 mL of conditioned medium for 2 h at room temperature. After washing, the arrays were incubated with biotinylated antibody cocktail at room temperature for 2 h, and then with Cy3 equivalent dye‐streptavidin for another 1 h at room temperature. Detection was performed according to the manufacturer's instructions.

### 2.10 Reverse transcription polymerase chain reaction (RT‐PCR)

Total RNA was extracted using the RNeasy^®^ Mini Kit (250) (QIAGEN, Hilden, Germany). Complementary DNA (cDNA) was synthesised from 1 μg of isolated total RNA by a high capacity cDNA reverse transcription kit (Thermo Fisher Scientific) in a final volume of 20 μL. The cDNA was subjected to real‐time PCR with the primers (Sigma‐Aldrich) as listed in Table S4.

PCR reactions were carried out in triplicate wells on ABI^®^ 7500 system using power SYBR Green master mix (Thermo Fisher Scientific). The PCR reaction for each well included 5 μL FastStart Essential DNA green master (Roche, Basel, Switzerland), 0.2 μL of each primer (20 μm) and 4.6 μL cDNA and 40 cycles (95 °C/15 s and 60 °C/30 s) of amplification were performed.

### 2.11 Western blots

Standard Western blots were undertaken using RIPA lysates as previously described [24].

### 2.12 Animals

Male immunodeficient NOD‐SCID mice (8 weeks old) purchased from Animal Resources Centre (Canning Vale, West Australia, Australia) were maintained in isolated ventilated cages under specific pathogen‐free conditions in a temperature‐ and humidity‐controlled, 12 h dark/light environment at the Monash Animal Research Platform (MARP), Monash University. The animals had free access to tap water and standard pellet food. Their health status was monitored daily. All procedures involving mice were conducted in accordance with the National Health and Medical Research Council (NHMRC) code for the use and care of animals for scientific purposes and the study was approved by the Monash University Animal Ethics Committee (Project ID: 21163).

### 2.13 Sub‐renal graft experiment

A total of 40 NOD‐SCID mice (8 mice per group) were used. Tissue recombinants consisting of 50 μL collagen gels that contain mixtures of cells (PC3 ± WPMY‐1 cells, 10^5^ ± 10^5^ cells total) were sub‐renally grafted under the kidney capsule of host mice. Each animal was implanted with 1–4 tissue recombinants placed on the left and/or right kidney. At the time of graft insertion surgery, a 5 mm silastic testosterone pellet was also inserted subcutaneously to help mimic the circulating testosterone levels of a human male.

Mice were sacrificed after 8 weeks by cervical dislocation. During autopsy, the xenograft tumours under the kidney capsules were resected for histological analysis. Tumour size was measured with a calliper. Tumour volume was calculated using the following formula: (long length × short length × width).

### 2.14 Statistical analysis

graphpad prism 9.0 (GraphPad Software, San Diego, CA, USA) was used for statistical calculations. For all comparisons between two groups, *t*‐tests were performed and *P* value and standard deviation of the mean (SD) were reported. For all comparisons among more than two groups, one‐way ANOVA was performed and *P* values and SD were reported. Results from all *in vitro* assays are representatives of at least three independent biological replicate experiments unless otherwise specified.

## 3 Results

### 3.1 Proteomic characterisation of co‐culture secretomes

#### 3.1.1 Overview

**Fig. 1:**
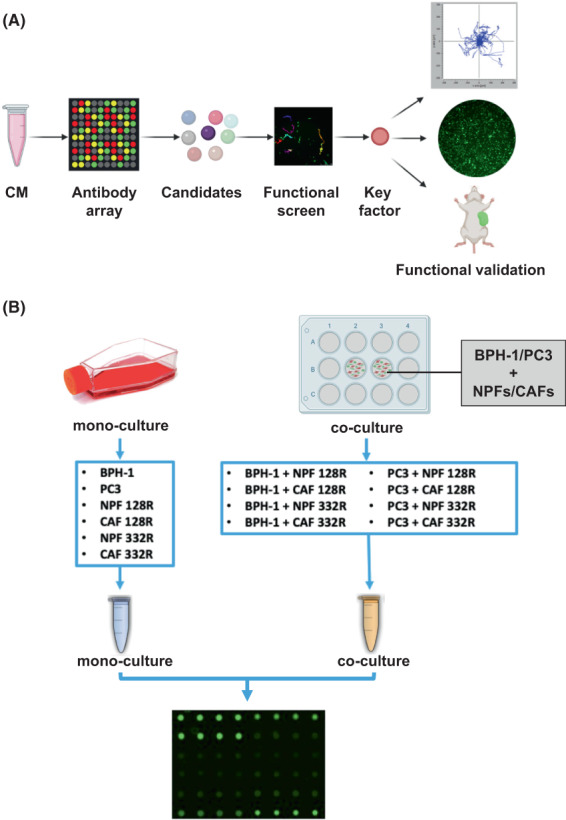
Experimental workflows. (A) Overall workflow for the project (Created with biorender software, BioRender, Toronto, Ontario, Canada). (B) Conditioned media (CM) preparation for antibody array analysis. Flow chart indicating how mono‐ and co‐culture conditioned media were prepared and analysed. CAF, cancer‐associated fibroblast; NPF, non‐malignant prostate fibroblast.

In order to characterise intercellular communication between prostate epithelial/cancer cells and normal or cancer‐associated fibroblasts (NPFs and CAFs, respectively), we employed an integrative approach involving three steps: (a) characterisation of mono‐ and co‐culture secretomes via antibody arrays; (b) functional interrogation of high priority candidates using a siRNA screen; and (c) a detailed characterisation of a lead candidate using *in vitro* and *in vivo* pre‐clinical models. The overall approach is summarised in Fig. 1A.

### 3.2 Profiling of conditioned media using a custom antibody array

Benign prostate epithelial cells (BPH‐1) or prostate cancer cells (PC3) were co‐cultured with CAFs or NPFs from 2 pairs of primary patient‐matched fibroblasts (NPF/CAF 128R, NPF/CAF 332R; Table S1) derived from radical prostatectomy specimens [21, 22]. The co‐culture conditioned medium, together with corresponding mono‐culture conditioned media, was collected and then assayed using antibody arrays that covered 200 human inflammation factors, growth factors, chemokines, cytokines and receptors (Fig. 1B, Table S5).

### 3.3 Secretome characterisation for prostate cell mono‐cultures

In total, 142 proteins were identified in the analysis of mono‐culture conditioned media (Fig. S1A). The secretion pattern of prostate epithelial/cancer cells was distinct from that of prostate fibroblasts in a number of ways. For example, DKK‐1, ErbB3 (soluble receptor, cleaved extracellular domain), Lipocalin‐2 and CEACAM‐1 were expressed at higher levels in conditioned media from BPH‐1 and PC3 cells compared to the primary fibroblasts. On the other hand, CCL11 (Eotaxin), HGF, TIMP‐1 and MCP‐1 (CCL2) were secreted more by the fibroblasts compared to prostate epithelial/cancer cells (Fig. S1B).

Different secretion patterns were also observed in the conditioned media between prostate epithelial and cancer cells (Fig. S1C). Secreted factors such as MIF, IL‐6, CD40L, FST, SDF‐1α and IL‐8 were expressed at higher levels in conditioned medium from PC3 cancer cells. In contrast, some factors were present at a higher level in BPH‐1 conditioned medium, including activin A, PAI‐1 and IGFBP‐3.

In addition, different secretion patterns were also observed between CAFs and NPFs (Fig. S1D). Examples of secreted factors expressed at higher levels in NPFs versus CAFs were EG‐VEGF, TGFα, GCP‐2, FGF‐4, BMP‐4, BMP‐5, IGFBP‐4, HGF (data shown in Fig. S1B) and PF4. In contrast, other secreted factors were presented at higher levels in CAF versus NPF conditioned media, such as IGFBP‐2, IL‐12p40, TNF‐β and MCSF.

While most expression patterns were consistent between pairs of NPFs or CAFs, some differences were observed (Fig. S1E). For example, IP‐10 (CXCL10) exhibited a very high level in CAF 128R compared to CAF 332R; IL‐8 was high in NPF 128R compared to NPF 332R. Differential expression was also observed for uPAR, IL‐16, Endoglin, IL‐9, PDGF‐AB and TIMP‐2.

### 3.4 Comparison of mono‐ and co‐culture secretomes

The antibody arrays were also used to characterise how conditioned media from co‐cultures of BPH‐1/PC3 with either NPFs or CAFs differed from the corresponding mono‐cultures. Unsupervised clustering of secretome patterns from the different cultures resulted in segregation of co‐cultures and mono‐cultures (Fig. S2).

While heat maps are a conventional approach to present different ‘–omics’ data, in order to resolve the complex datasets to the greatest extent, an X‐plot approach was developed (Fig. S3). Here, each dot in the X‐plot represents a secreted factor, whose x‐coordinate value indicates expression value in mono‐culture conditioned media and y‐coordinate value indicates expression value in co‐culture conditioned media. For example, in Fig. S3, quadrant I represents the comparison of BPH‐1 + NPF 128R co‐culture with BPH‐1 mono‐culture and quadrant II shows BPH‐1 + NPF 128R co‐culture versus NPF 128R mono‐culture. The red dots represent factors with an increase of expression in co‐culture, as they are closer to the y‐axis; the blue dots denote the secreted factors decreasing in co‐culture, as they are closer to the x‐axis. Accordingly, the comparison between BPH‐1 + CAF 128R co‐culture and mono‐culture is plotted in quadrant III and IV in the similar way (Fig. S3).

### 3.5 The secretome of prostate epithelial cell/fibroblast co‐cultures

**Fig. 2:**
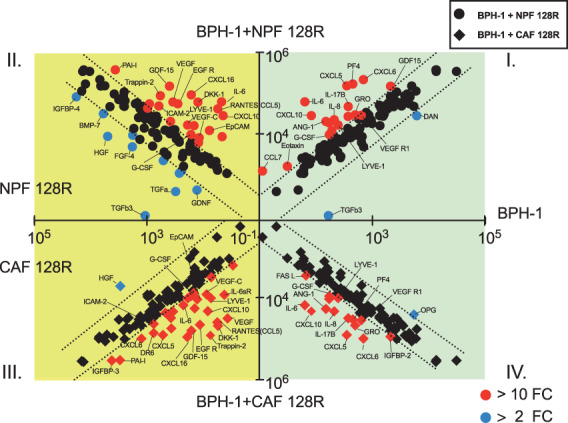
X‐plot approach to visualise how secretomes of co‐cultures of BPH‐1 with NPFs or CAFs differ from corresponding mono‐cultures. Scatter plots with log10 transformed axes, where up‐regulated cytokine/chemokines (red dots) were selected using a stringent cut‐off value of FC > 10 (protein level in co‐culture/corresponding mono‐culture), and decreased cytokine/chemokine (blue dots) were selected by a cut‐off value of > 2 FC (protein level in mono‐culture/corresponding co‐culture). Green background: BPH‐1 cytokine/chemokine secretion, yellow background: fibroblast cytokine/chemokine secretion. The primary screen was undertaken with one biological replicate. FC, fold change; CAF, cancer‐associated fibroblast; NPF, non‐malignant prostate fibroblast.

A total of 116 secreted factors were detected and included in the analysis. The secretomes of BPH‐1 co‐cultured with NPF/CAF 128R and NPF/CAF 332R are visualised in Fig. 2 and Fig. S4, respectively. Of note, the levels of some proteins were increased in co‐culture compared to all mono‐cultures, such as CXCL10, IL‐6, LYVE‐1 and GDF‐15 (Fig. 2 and Fig. S4A). CXCL10, a known chemoattractant that exhibits anti‐tumour activity [25, 26], was particularly prominent, with its expression up‐regulated in the co‐cultures with either NPFs or CAFs by up to three orders of magnitude (Fig. S4B). Another example with high expression is GDF15 (growth differentiation factor 15), a member of the transforming growth factor beta superfamily involved in regulating apoptosis, cell growth and carcinogenesis [27] (Fig. S4C).

In addition to proteins that showed increased levels in the co‐culture compared to all mono‐cultures, some secreted factors were expressed at different levels in co‐cultures with NPFs versus CAFs, including IGFBP‐3, IGFBP‐4, DR6, SCF and activin A in BPH‐1 co‐cultures with NPF/CAF 128R (Fig. S5A) and NPF/CAF 332R (Fig. S5B). Death receptor 6 (DR6, a cleaved extracellular domain in this antibody array), also known as tumour necrosis factor receptor superfamily member 21(TNFRSF21), is a cell surface receptor of the tumour necrosis factor receptor superfamily which activates the JNK and NF‐κB pathway [28]. DR6 was expressed at a relatively high level in BPH‐1 and low levels in fibroblast mono‐culture conditioned media respectively, but its level was markedly higher in BPH‐1 + CAFs co‐culture conditioned medium versus BPH‐1 + NPFs co‐culture conditioned medium (Fig. S5C).

### 3.6 The secretome of prostate cancer cell/fibroblast co‐cultures

**Fig. 3:**
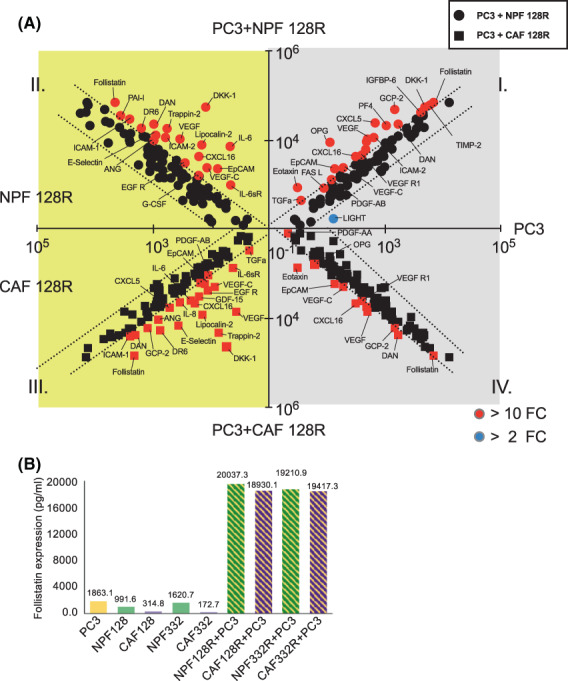
Secretome comparison between co‐cultures of PC3 with NPFs or CAFs and corresponding mono‐cultures. (A) X plots where up‐regulated cytokine/chemokines (red dots) were selected using a stringent cut‐off value of FC > 10 (protein level in co‐culture/corresponding mono‐culture), and decreased cytokine/chemokines (blue dots) were selected by a cut‐off value of > 2 FC (protein level in mono‐culture/corresponding co‐culture). Grey background: PC3 cytokine/chemokine secretion, yellow background: fibroblast cytokine/chemokine secretion. (B) FST expression level in co‐cultures and mono‐cultures. The primary screen was undertaken with one biological replicate. FC, fold change; FST, follistatin; CAF, cancer‐associated fibroblast; NPF, non‐malignant prostate fibroblast.

Ninety‐six proteins were detected and included in the analysis of the conditioned media from co‐culture versus mono‐culture. Compared to BPH‐1, a lower number of proteins decreased expression in co‐culture and most of the changes manifested in proteins that increased in expression (Fig. 3A and Fig. S6). This was particularly evident in 128R co‐culture, which revealed only one protein [LIGHT, also known as tumour necrosis factor superfamily member 14 (TNFSF14)] that markedly decreased in expression compared to PC3 mono‐culture (Fig. 3A). Similar to the BPH‐1 co‐culture, some proteins were up‐regulated in the co‐cultures compared to all corresponding mono‐cultures, including DAN, FST, EpCAM, VEGF and CXCL16 (Fig. 3A and Fig. S6). For example, FST was expressed at low levels in all mono‐cultures, but it showed an increase in the co‐cultures of between one and two orders of magnitude (Fig. 3B).

Other secreted factors exhibited contrasting changes in expression when PC3 cells were co‐cultured with either NPFs or CAFs, for example PDGF‐AA, TGFα and CXCL4 (Fig. S7A and S7B). Transforming growth factor alpha (TGFα) is a ligand for the EGF receptor and has been associated with many types of cancer [29, 30]. NPFs secreted a high level of TGFα, while the CAFs and PC3s did not (Fig. S7C). However, after co‐culture, the PC3 + CAF co‐cultures expressed a substantial level of TGFα, much higher than CAF and PC3 mono‐cultures, but still lower than PC3 + NPF co‐cultures.

Overall, the antibody array analysis revealed distinct secretion patterns where proteins were markedly increased in co‐cultures versus corresponding mono‐cultures (e.g. CXCL10, IL‐6 in BPH‐1 co‐culture and CXCL16, DKK‐1, FST in PC3 co‐culture). In addition, it identified differential effects of CAF versus NPF co‐culture (e.g. on DR6). Importantly, these data highlight how intercellular communication between the cell types affects the co‐culture secretome so that levels of particular secreted factors in the co‐culture cannot be predicted based on the mono‐cultures.

### 3.7 Functional evaluation of secreted factors via a custom siRNA screen

**Fig. 4:**
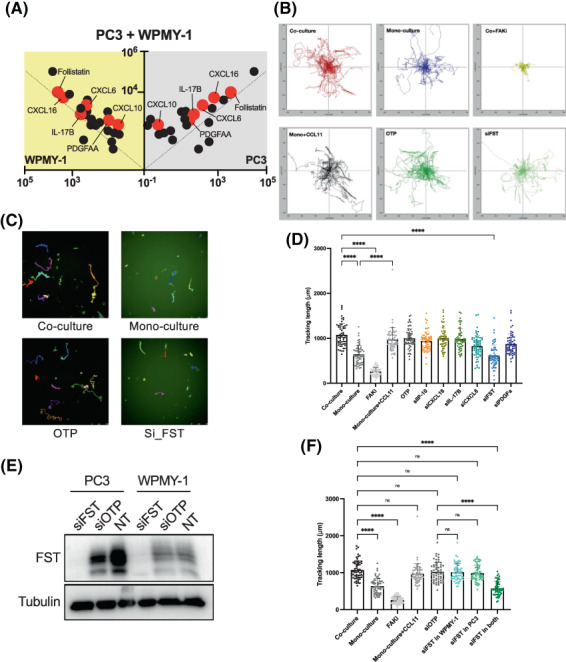
A random cell migration assay identifies FST as a critical regulator in the co‐culture system. (A) Half X plot of cytokine/chemokines in the secondary array screen. Grey background: PC3 cytokine/chemokine secretion, yellow background: WPMY‐1 cytokine/chemokine secretion. Red dots indicate the six candidates. The secondary screen was undertaken with one biological replicate. (B) Trajectories of PC3‐GFP cells in the co‐culture system when FST was knocked down in both PC3‐GFP and WPMY‐1 cells. (C) Examples of dragon tails display showing single PC3‐GFP cell migration tracks in which temporal changes in cell location are indicated as coloured lines (last frames of the time‐lapse movies). (D) Quantification of the accumulative migrated distance which PC3‐GFP cells travelled in mono‐culture and in co‐culture with WPMY‐1 when different targets were knocked down in both PC3‐GFP and WPMY‐1 cells (representative of *n* = 3). (E) Knockdown efficiency in both PC3 and WPMY‐1 cells was confirmed by western blot (representative of *n* = 3). The targets were knocked down by transfection with corresponding siRNA with ON‐TARGETplus (OTP) as the negative control. (F) FST knockdown in both cell types in the co‐culture system is required to impair PC3 cell migration. Quantification of the accumulative migrated distance which PC3‐GFP cells travelled when FST was knocked down individually in PC3‐GFP or WPMY‐1 cells as well as in both cell types in the co‐culture system (*n* = 3). Each data point represents a single cell that has been analysed in the time‐lapse movies. Data are presented as mean ± SD; one‐way ANOVA, Tukey's multiple comparisons; ns, not significant, ****P < 0.0001. FST, follistatin; FAKi, focal adhesion kinase inhibitor; NT, non‐treatment.

Next, we set out to functionally characterise specific factors in the co‐culture setting. Due to the limited lifespan of the primary fibroblasts and difficulties in manipulating them by transfection, we decided to utilise WPMY‐1, an immortalised prostate stromal cell line, in combination with PC3s in functional screens. In order to implement this model, it was first necessary to undertake a secondary antibody array screen to confirm that changes observed in co‐cultures of PC3s with NPFs or CAFs were observed with WPMY‐1 cells. This was undertaken using a customised antibody array that detected 24 secreted proteins (Table S6), selected from the primary screen on the basis of novelty; biological function; known relationship to cancer; and tractability, including availability of validated reagents. Upon comparing PC3/WPMY‐1 co‐cultures with the corresponding mono‐cultures, many proteins exhibited similar trends to the primary screen, including CXCL1, CCL2, CCL5, IL‐8, CXCL10 and FST (Fig. S8A–J, Table S6). Then, we excluded proteins based on (a) a different trend in the WPMY‐1 co‐culture system compared to the primary screen, despite their significant biological roles in tumourigenesis and progression (BDNF [31], IL‐6sR [32], TGFα [33] and IL‐6) (Table S6), or (b) lack of novelty, reflecting well‐characterised roles in cancer, such as CXCL1, CCL2, CCL5, IL‐6 and IL‐8. This led to the selection of six proteins (CXCL10, CXCL16, CXCL6, FST, PDGFAA and IL‐17B), all up‐regulated in co‐culture conditioned media compared to corresponding mono‐cultures (Fig. 4A, Fig. S8E–J, Table S6).

Then, the six candidate targets were subjected to a functional screen based on random cell migration. Briefly, targets were individually knocked down by corresponding siRNA in both PC3‐GFP and WPMY‐1 cells, then the cells were co‐cultured and PC3‐GFP migration characteristics assayed in terms of accumulative migrated distance (the total distance that one single cell travels within a defined time; Fig. 4B,C). Human recombinant CCL11 protein, a known pro‐migratory cytokine [34], was added to PC3‐GFP mono‐culture as the positive control for migratory enhancement and the selective FAK inhibitor (FAKi) PF‐562271 [35] was added to the co‐culture as the positive control for migratory inhibition (Fig. 4B,D, Fig. S9A,B). After 24 h random migration, co‐culture with WPMY‐1 cells significantly increased the accumulative migrated distance of PC3‐GFP cells compared to PC3‐GFP culture alone. The motility of PC3‐GFP cells was markedly impaired when the FAKi was added to the co‐culture system. In addition, their migration in mono‐culture was significantly increased by human recombinant CCL11 protein. When each of the six targets were individually knocked down in both cell types in the co‐culture, it was found that FST knockdown (Fig. 4E and Fig. S9C) significantly inhibited PC3‐GFP cell migration in co‐culture, whereas this effect was not seen with the other targets (Fig. 4B–D, Fig. S9A,B). These data demonstrate that expression of FST by PC3 cells and/or WPMY‐1 fibroblasts is required for WPMY‐1 cells to enhance PC3 cell migration.

### 3.8 Characterisation of FST as a key regulator of intercellular communication between prostate cancer cells and co‐cultured fibroblasts

To characterise which cell type in the co‐culture system was producing the FST responsible for the pro‐migratory effect, FST was knocked down in either PC3 or WPMY‐1 cells or both cell types, and random cell motility of the PC3‐GFP cells assayed in co‐cultures. The motility of the PC3‐GFP cells was only significantly inhibited when FST was knocked down in both cell types (Fig. 4F, Fig. S9D,E), not in one cell type only. This indicates that FST is produced by both PC3 and WMPY‐1 cells, so that blocking its function in promoting cell migration requires its knock down in both cell types.

**Fig. 5:**
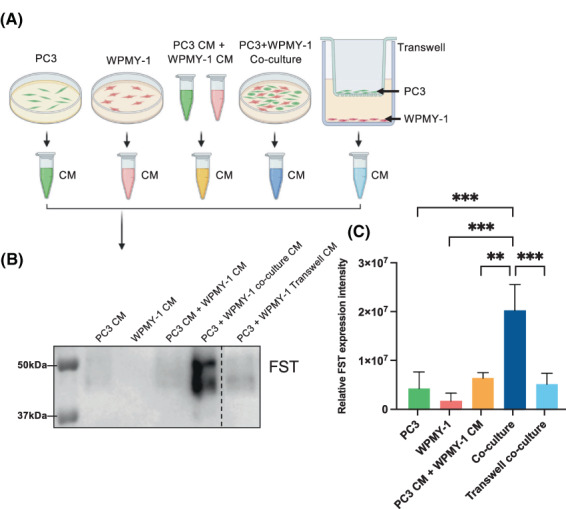
Characterisation of the requirements for FST production indicates direct co‐culture is essential for promoting FST secretion. (A) Schematic illustrating the different culture conditions (Created with biorender software). (B) Levels of FST in the conditioned media from PC3 and WPMY‐1 mono‐cultures, in combined mono‐culture and from co‐cultures (direct and Transwell) were analysed by western blot. The same volumes of conditioned media were loaded for each sample. The dashed line represents where the blot was cut. (C) Quantification of western blots by densitometry (*n* = 3). Data are presented as mean ± SD; one‐way ANOVA, Tukey multiple comparisons; **P < 0.01, ***P < 0.001. CM, conditioned media; FST, follistatin.

In order to characterise the requirements for FST production in terms of cell–cell proximity, we analysed conditioned media from mono‐cultures, direct co‐culture, and Transwell co‐culture of PC3 and WPMY‐1 cells by western blot analysis (Fig. 5A). Strikingly, a marked increase in FST expression was only observed when the cells were directly co‐cultured, indicating that the two cell types must be in close proximity to promote enhanced FST production (Fig. 5B,C).

**Fig. 6:**
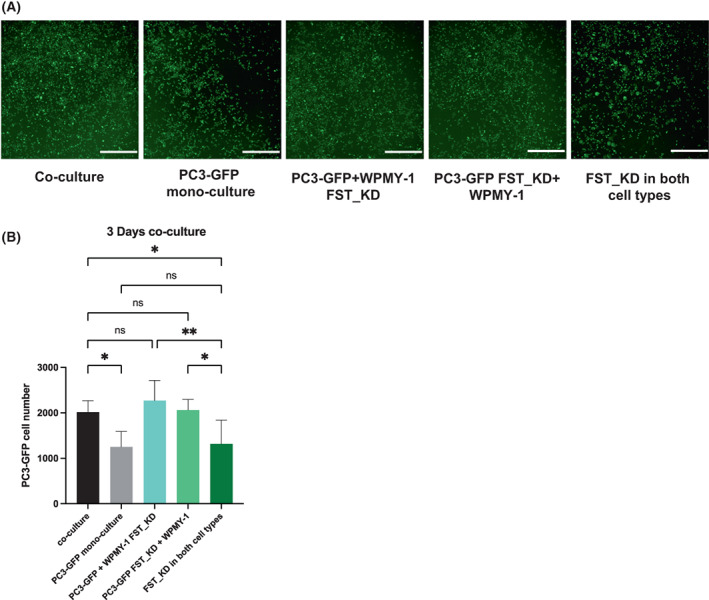
FST knockdown by CRISPRi in the co‐culture system impairs the proliferation of prostate cancer cells. FST was knocked down in PC3‐GFP and WPMY‐1 cells by transfected sgRNA specifically targeting FST. (A) Representative images of wildtype/knockdown PC3‐GFP cells cultured alone or co‐cultured with wildtype/knockdown WPMY‐1 cells after 3 days. (B) Quantification of green fluorescence cells in mono‐culture and co‐culture, after 3 days co‐culture (*n* = 3). Data are presented as mean ± SD; one‐way ANOVA, Tukey multiple comparisons; ns, not significant, *P < 0.05, **P < 0.01. Scale bar: 1 mm (A). KD, knockdown; FST, follistatin.

Determination of the role of FST in regulating longer term biological endpoints, such as proliferation, as well as tumour growth *in vivo*, necessitated generation of stable FST knockdown cell lines by CRISPRi. The knockdown efficiency in both PC3 and WPMY‐1 cells was validated by western blot. Two out of five sgRNAs exhibited strong knockdown efficiency (Fig. S10A–C). The cells were then subjected to random cell migration assays, where consistent with the data using siRNA (Fig. 4F), knockdown in both cell types in the co‐culture system was required to significantly reduce the accumulative moving distance of the PC3‐GFP cells in co‐culture (Fig. S11A–C). The same result was observed for cell displacement (maximum distance from start point during the migration; Fig. S11D). Rescue experiments were also undertaken to validate the role of FST and confirm that the data did not reflect off‐target effects. Addition of human recombinant FST (hrFST) rescued the decreased accumulative moving distance and cell displacement of these cells in the double knockdown co‐cultures (Fig. S12A–D). We then determined the role of FST in regulating cell proliferation. Co‐culture of PC3‐GFP cells with WPMY‐1 fibroblasts significantly increased the proliferation of the former (Fig. 6). However, similar to the cell migration result, this effect was lost upon knockdown of FST in both PC3‐GFP and WPMY‐1 cells, but not in only one of these cell types (Fig. 6A,B). Similar to the migration result, addition of hrFST rescued the decreased proliferation of PC3‐GFP cells in co‐culture when FST was knocked down in both cell types (Fig. S13A–D).

**Fig. 7:**
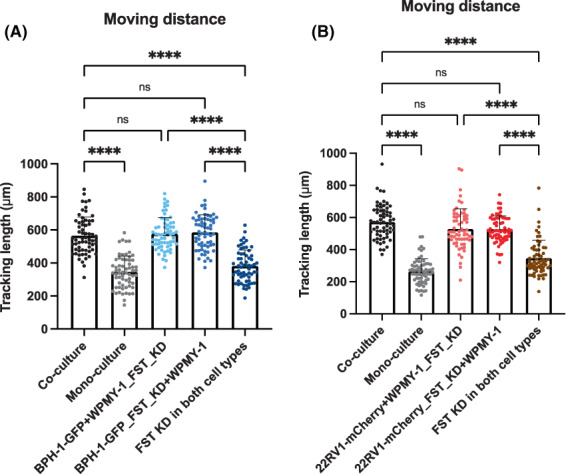
FST regulates migration of additional prostate epithelial and prostate cancer cell lines in co‐culture with fibroblasts. (A, B) Quantification of the accumulative migrated distance which BPH‐1‐GFP (A) or 22RV1‐mCherry (B) cells travelled when FST was knocked down in one or both cell types in the co‐culture system (*n* = 3). Each data point represents a single cell that has been analysed in the time‐lapse movies. Data are presented as mean ± SD; one‐way ANOVA, Tukey's multiple comparisons; ns, not significant, ****P < 0.0001. KD, knockdown; FST, follistatin.

In order to ascertain the broader applicability of these findings, we interrogated the role of FST in other prostate epithelial (BPH‐1) or prostate cancer (DU145, LNCaP and 22RV1) cell lines. Using siRNA, we obtained strong FST knockdown efficiencies in BPH‐1 and 22RV1 cells (Fig. S14A,B). The migration of these two cell lines was then assayed in mono‐ and WPMY‐1 co‐cultures, using the same single and double knockdown approach as described previously (Fig. 4). The results were consistent with the effects for PC3‐GFP + WPMY‐1 co‐culture, in that co‐culture with WPMY‐1 cells significantly increased the motility of BPH‐1‐GFP and 22RV1‐mCherry cells compared to their mono‐cultures. Likewise, the migratory abilities of BPH‐1‐GFP and 22RV1‐mCherry cells in WPMY‐1 co‐culture decreased only when FST was knocked down in both cell types (Fig. 7, Fig. S15A,B).

In summary, these results indicate that a marked increase in FST production occurs when prostate cancer cells are co‐cultured with prostate fibroblasts, both cell types generate functional FST and this factor promotes both proliferation and migration of co‐cultured prostate cancer cells.

### 3.9 Determination of the role of FST in regulating tumour growth

**Fig. 8:**
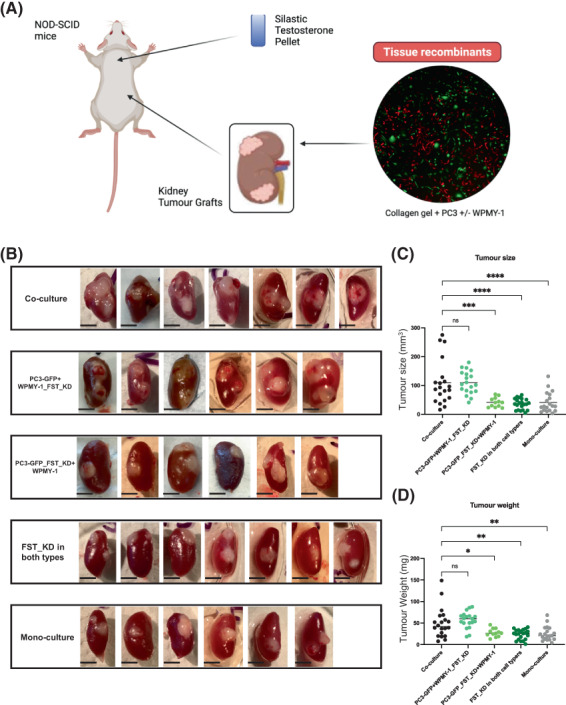
FST knockdown in prostate cancer cells impairs tumour growth in a mouse xenograft model. (A) Schematic representation of the experimental procedure (Created with biorender software). (B) Representative images of sub‐renal grafts resulting from injection of different cell mixtures. Each image represents one sub‐renal graft from one mouse. (C, D) Quantification of tumour size (C) and weight (D) at the end point (8 weeks) after resection. Each data point represents a single sub‐renal graft (*n* = 8 per group). Data are presented as mean ± SD; one‐way ANOVA, Dunnett's multiple comparisons; ns, not significant, *P < 0.05, **P < 0.01, ***P < 0.001, ****P < 0.0001. Scale bar: 3 mm (B). KD, knockdown; FST, follistatin.

To the best of our knowledge, the role of FST in prostate tumour growth has not been characterised using a mouse xenograft model. To expand on the *in vitro* findings, a mouse sub‐renal graft model was employed to test whether FST KD could have an impact on tumour growth following implantation of PC3/WPMY‐1 cell recombinants (Fig. 8A).

Importantly, co‐injection with fibroblasts significantly enhanced both tumour size and weight (Fig. 8B–D). In addition, FST knockdown in the WPMY‐1 cells did not affect these parameters. However, unlike the *in vitro* assays for cell proliferation and migration, knockdown of FST in either the PC3 cells, or both cell types, reduced tumour growth to a similar degree (Fig. 8B–D). This likely reflects differences between 2D co‐culture *in vitro* and 3D growth *in vivo*, and the presence of the host environment. It also indicates that the FST signal *in vivo* is predominantly from the PC3 cancer cells, so that depletion of FST in the PC3 cells leads to decreased tumour growth, and that in this model, the WPMY‐1 cells promote tumour growth by a FST‐independent mechanism.

## 4 Discussion

The current study explored the intercellular communication between prostate stromal fibroblasts and cancer cells, which is a well‐documented driver of tumour progression in prostate cancer [7, 8]. By systematically profiling the conditioned media from both individual cultures or co‐culture of prostate fibroblasts and prostate epithelial or cancer cells, marked changes in co‐culture secretomes were revealed, highlighting specific TME‐derived secreted factors as candidate therapeutic targets. Additionally, the study identified the critical role of the secreted glycoprotein FST, characterising it for the first time to our knowledge in the context of prostate cancer/PE crosstalk with fibroblasts, and demonstrating that it enhances prostate cancer cell proliferation and migration *in vitro* and also tumour growth *in vivo*.

Characterisation of secreted factors in co‐cultures, rather than the corresponding mono‐cultures, is a relatively uncharted area, and the co‐culture may only be compared to one cell type, as in a recent study on IL‐6 in gastric cancer [36]. A key feature of our study is the discovery of candidate secreted factors through an antibody array approach in which we not only characterise the secretomes from prostate epithelial/cancer cells, or CAF and NPF mono‐cultures, but also compare them with those from corresponding co‐cultures. This determined that while the different mono‐cultures could exhibit distinct secretion patterns, the secretomes of the co‐cultures could be completely different, and this was not a simple additive effect. Consequently, the levels of particular secreted factors in the co‐culture cannot be predicted based on the mono‐cultures since intercellular communication between the cell types affects the co‐culture secretome. This communication may be mediated via soluble or ECM factors, or via direct cell–cell contact. In our model, cell–cell contact, or at least very close cell proximity, was necessary to enhance the production of FST. Similar findings have been reported in other cell systems. For example, chronic inflammatory diseases feature massive up‐regulation of IL‐1 and TNF induced by direct cellular contact of T cells and monocytes/macrophages [37] and direct co‐culture of splenocytes and adipocytes leads to a greater increase in IL‐6 and MCP‐1 levels compared to indirect co‐culture and individual culture [38]. Furthermore, direct cell–cell contact of pancreatic cancer cells and fibroblasts induces calcium oscillations, NF‐κB activation, and activin A secretion, leading to increased EMT of cancer cells [39].

Follistatin is associated with the development, progression and metastasis of various cancers. The primary function of FST is as an antagonist of specific TGF‐β superfamily proteins, including activins (activin A, B and C), bone morphogenetic proteins (e.g. BMP‐4, BMP‐7, BMP‐11) [40, 41], Myostatin [40] and TGF‐β3 [42]. FST has a high affinity for activin A [18], and increasing evidence reveals that dysregulation of the FST/activin A system could lead to alterations of the normal homeostasis of prostate tissue and cause development and progression of prostate cancer [43, 44, 45, 46]. For example, activin A's inhibition of prostate cancer cell proliferation is completely reversed by FST [44, 47, 48]. Furthermore, serum levels of FST are increased in prostate cancer patients [19] and overexpression of FST occurs in tumours compared to normal prostate tissues [49], supporting a model where FST suppresses activin signalling‐mediated cell growth inhibition and promotes prostate cancer development and progression [50, 51]. However, recent data indicate that activin A signalling, and hence potentially FST, may play stage‐specific roles in prostate cancer, with non‐canonical activin A signalling maintaining epithelial quiescence in the normal prostate [45] but activin A positively regulating metastasis in pre‐clinical models and associating with poor prognosis in prostate cancer patients [46].

Despite high serum levels of FST associating with poor prognosis in hepatocellular carcinoma [52, 53] and high tumour FST also representing a negative prognostic factor in lung, ovarian and gastric cancer [49], survival data from the TCGA database indicates that high tumoural expression of FST does not correlate with altered disease‐free survival of prostate cancer patients (Fig. S16). However, it should be noted that the TCGA data correspond to FST mRNA not protein, and it's possible that FST expression is regulated at the post‐transcriptional level and/or at the level of secretion. In addition, it is unclear from the TCGA data whether gene expression relates to the cancer cells, CAFs, or both. Moreover, FST could have opposing effects at different stages of disease progression, as indicated for activin A.

In addition to FST, other factors also exhibited increased expression in co‐culture conditioned media, and although their knockdown did not reduce the migratory ability of PC3 cells in the functional screen, these factors might exhibit other biological roles. Three of them (CXCL10, CXCL6 and CXCL16) belong to the chemokine (C‐X‐C motif) ligand (CXCL) family, which are known to play key roles in inflammatory diseases, neoplastic transformation and tumour growth regulation [54]. CXCL10 increases the proliferation of mouse breast cancer stem cells and breast cancer cells, and is significantly associated with triple negative breast cancer versus HER2^+^, Luminal A and Luminal B breast cancer [55]. CXCL6 production is up‐regulated in aged prostate stroma and promotes proliferation of both prostate stromal fibroblasts and epithelium [56], and increased CXCL6 expression occurs in prostate cancers with high Notch1 levels [57]. In addition, CXCL16 can promote the proliferation of PC3 cells and high expression of CXCL16 correlates with high‐stage and high‐grade prostate cancer [58, 59]. The CXCR6‐CXCL16 axis also promotes docetaxel resistance through phosphorylation of GSK‐3β, NF‐κB and ERK1/2 [60], as well as angiogenesis in prostate cancer via AKT/mTOR‐mediated regulation of VEGF and IL‐8 [61]. Consequently, these factors exhibit crucial biological roles in cancer development and progression, and are likely to emerge as ‘hits’ in screens that incorporate other biological endpoints.

## 5 Conclusions

In summary, our work highlights potent intercellular communication between prostate stromal fibroblasts and epithelial/cancer cells that results in a reprogrammed combined secretome. It also reveals FST as a key factor involved in prostate cancer‐stroma interaction *in vitro* and *in vivo*, suggesting that the role of FST should be revisited in the context of different stages and mutational profiles of prostate cancer, which may provide insights from a precision oncology perspective and identify potential strategies for disease management.

## Conflict of interest

Renea A. Taylor declares research collaborations: Pfizer, Astellas, Zenith Epigenetics, AstraZeneca; all other authors indicate that there are no competing interests.

## Author contributions

Conceptualisation: RJD, RAT. Experiments and investigation: YW, KCC, BN. Formal analysis: YW, CC, ACC. Methodology: RJD, RAT, YW, KCC, BN, ACC, LGH. Visualisation: YW. Writing‐original draft: YW. Writing‐review & editing: YW, LGH, KCC, RAT, RJD.

## Supporting information


**Fig. S1.** Secretome of mono‐cultured prostate epithelial/cancer cells and patient‐derived fibroblasts.
**Fig. S2.** Secretome of co‐cultured prostate epithelial/cancer cells with NPFs/CAFs.
**Fig. S3.** Schematic of data visualisation by the X‐plot.
**Fig. S4.** X‐plot approach to visualise how secretomes of co‐cultures of BPH‐1 with NPFs or CAFs differ from corresponding mono‐cultures.
**Fig. S5.** X‐Plot highlighting proteins with differential expression between NPF and CAF co‐cultures.
**Fig. S6.** Secretome comparison between co‐cultures of PC3 with NPFs or CAFs and corresponding mono‐cultures.
**Fig. S7.** X‐Plot highlighting proteins with differential expression changes in PC3 co‐cultures with NPFs or CAFs.
**Fig. S8.** Expression levels of secreted factors in primary and secondary cytokine/chemokine array screens.
**Fig. S9.** A random cell migration assay identifies FST as a critical regulator in the co‐culture system.
**Fig. S10.** FST knockdown in WPMY‐1 and PC3‐GFP cells by CRISPRi.
**Fig. S11.** Stable knockdown of FST in both cell types by CRISPRi impairs migration of prostate cancer cells in co‐culture.
**Fig. S12.** Human recombinant FST rescues the decreased migration of prostate cancer cells in co‐culture caused by stable FST knockdown.
**Fig. S13.** Human recombinant FST rescues the impaired proliferation of prostate cancer cells in co‐culture mediated by stable FST knockdown.
**Fig. S14.** FST expression and knockdown in different prostate epithelial/cancer cell lines.
**Fig. S15.** FST regulates migration of additional prostate epithelial/cancer cell lines in co‐culture with fibroblasts.
**Fig. S16.** Relationship of tumoural FST expression to disease‐free survival of patients with prostate cancer.
**Table S2.** siRNA sequences used.
**Table S3.** Summary of sgRNA sequences and primers.
**Table S4.** Real‐time PCR primers.

**Table S1.** Clinicopathological features of tumours of origin for primary fibroblast cell lines.
**Table S5.** List of proteins for the 200 human antibody array analysis.
**Table S6.** Expression of targets in the first and secondary customised antibody array analysis.

## Data Availability

The authors confirm that all data underlying the findings are fully available without restriction.

## References

[1] Sung H , Ferlay J , Siegel RL , Laversanne M , Soerjomataram I , Jemal A , et al. Global cancer statistics 2020: GLOBOCAN estimates of incidence and mortality worldwide for 36 cancers in 185 countries. CA Cancer J Clin. 2021;71(3):209–49. 33538338 10.3322/caac.21660

[2] Ferlay J , Colombet M , Soerjomataram I , Mathers C , Parkin DM , Pineros M , et al. Estimating the global cancer incidence and mortality in 2018: GLOBOCAN sources and methods. Int J Cancer. 2019;144(8):1941–53. 30350310 10.1002/ijc.31937

[3] Quail DF , Joyce JA . Microenvironmental regulation of tumor progression and metastasis. Nat Med. 2013;19(11):1423–37. 24202395 10.1038/nm.3394 PMC3954707

[4] Junttila MR , de Sauvage FJ . Influence of tumour micro‐environment heterogeneity on therapeutic response. Nature. 2013;501(7467):346–54. 24048067 10.1038/nature12626

[5] Sun DY , Wu JQ , He ZH , He MF , Sun HB . Cancer‐associated fibroblast regulate proliferation and migration of prostate cancer cells through TGF‐beta signaling pathway. Life Sci. 2019;235:116791. 31465732 10.1016/j.lfs.2019.116791

[6] Ippolito L , Morandi A , Taddei ML , Parri M , Comito G , Iscaro A , et al. Cancer‐associated fibroblasts promote prostate cancer malignancy via metabolic rewiring and mitochondrial transfer. Oncogene. 2019;38(27):5339–55. 30936458 10.1038/s41388-019-0805-7

[7] Zhang Z , Karthaus WR , Lee YS , Gao VR , Wu C , Russo JW , et al. Tumor microenvironment‐derived NRG1 promotes antiandrogen resistance in prostate cancer. Cancer Cell. 2020;38(2):279–96. e9. 32679108 10.1016/j.ccell.2020.06.005 PMC7472556

[8] Wu Z , Shi J , Lai C , Li K , Li K , Li Z , et al. Clinicopathological significance and prognostic value of cancer‐associated fibroblasts in prostate cancer patients. Urol Oncol. 2021;39(7):433 e17–23. 10.1016/j.urolonc.2021.05.004 34112577

[9] De Wever O , Demetter P , Mareel M , Bracke M . Stromal myofibroblasts are drivers of invasive cancer growth. Int J Cancer. 2008;123(10):2229–38. 18777559 10.1002/ijc.23925

[10] Bartoschek M , Oskolkov N , Bocci M , Lovrot J , Larsson C , Sommarin M , et al. Spatially and functionally distinct subclasses of breast cancer‐associated fibroblasts revealed by single cell RNA sequencing. Nat Commun. 2018;9(1):5150. 30514914 10.1038/s41467-018-07582-3 PMC6279758

[11] Pistore C , Giannoni E , Colangelo T , Rizzo F , Magnani E , Muccillo L , et al. DNA methylation variations are required for epithelial‐to‐mesenchymal transition induced by cancer‐associated fibroblasts in prostate cancer cells. Oncogene. 2017;36(40):5551–66. 28581528 10.1038/onc.2017.159

[12] Toullec A , Gerald D , Despouy G , Bourachot B , Cardon M , Lefort S , et al. Oxidative stress promotes myofibroblast differentiation and tumour spreading. EMBO Mol Med. 2010;2(6):211–30. 20535745 10.1002/emmm.201000073 PMC3377319

[13] Cioni B , Nevedomskaya E , Melis MHM , van Burgsteden J , Stelloo S , Hodel E , et al. Loss of androgen receptor signaling in prostate cancer‐associated fibroblasts (CAFs) promotes CCL2‐ and CXCL8‐mediated cancer cell migration. Mol Oncol. 2018;12(8):1308–23. 29808619 10.1002/1878-0261.12327 PMC6068356

[14] Maxwell PJ , Neisen J , Messenger J , Waugh DJ . Tumor‐derived CXCL8 signaling augments stroma‐derived CCL2‐promoted proliferation and CXCL12‐mediated invasion of PTEN‐deficient prostate cancer cells. Oncotarget. 2014;5(13):4895–908. 24970800 10.18632/oncotarget.2052 PMC4148108

[15] Samain R , Brunel A , Douche T , Fanjul M , Cassant‐Sourdy S , Rochotte J , et al. Pharmacologic normalization of pancreatic cancer‐associated fibroblast Secretome impairs Prometastatic cross‐talk with macrophages. Cell Mol Gastroenterol Hepatol. 2021;11(5):1405–36. 33482394 10.1016/j.jcmgh.2021.01.008 PMC8024982

[16] Jia C , Wang G , Wang T , Fu B , Zhang Y , Huang L , et al. Cancer‐associated fibroblasts induce epithelial‐mesenchymal transition via the transglutaminase 2‐dependent IL‐6/IL6R/STAT3 axis in hepatocellular carcinoma. Int J Biol Sci. 2020;16(14):2542–58. 32792856 10.7150/ijbs.45446 PMC7415430

[17] Tortoriello DV , Sidis Y , Holtzman DA , Holmes WE , Schneyer AL . Human follistatin‐related protein: a structural homologue of follistatin with nuclear localization. Endocrinology. 2001;142(8):3426–34. 11459787 10.1210/endo.142.8.8319

[18] Cash JN , Angerman EB , Keutmann HT , Thompson TB . Characterization of follistatin‐type domains and their contribution to myostatin and activin a antagonism. Mol Endocrinol. 2012;26(7):1167–78. 22593183 10.1210/me.2012-1061 PMC3385792

[19] Tumminello FM , Badalamenti G , Fulfaro F , Incorvaia L , Crescimanno M , Flandina C , et al. Serum follistatin in patients with prostate cancer metastatic to the bone. Clin Exp Metastasis. 2010;27(8):549–55. 20623366 10.1007/s10585-010-9344-x

[20] Tang Z , Li C , Kang B , Gao G , Li C , Zhang Z . GEPIA: a web server for cancer and normal gene expression profiling and interactive analyses. Nucleic Acids Res. 2017;45(W1):W98–W102. 28407145 10.1093/nar/gkx247 PMC5570223

[21] Lawrence MG , Taylor RA , Toivanen R , Pedersen J , Norden S , Pook DW , et al. A preclinical xenograft model of prostate cancer using human tumors. Nat Protoc. 2013;8(5):836–48. 23558784 10.1038/nprot.2013.043

[22] Taylor RA , Toivanen R , Frydenberg M , Pedersen J , Harewood L , B. Australian Prostate Cancer , et al. Human epithelial basal cells are cells of origin of prostate cancer, independent of CD133 status. Stem Cells. 2012;30(6):1087–96. 22593016 10.1002/stem.1094

[23] Mu P , Zhang Z , Benelli M , Karthaus WR , Hoover E , Chen CC , et al. SOX2 promotes lineage plasticity and antiandrogen resistance in TP53‐ and RB1‐deficient prostate cancer. Science. 2017;355(6320):84–8. 28059768 10.1126/science.aah4307 PMC5247742

[24] Brummer T , Schramek D , Hayes VM , Bennett HL , Caldon CE , Musgrove EA , et al. Increased proliferation and altered growth factor dependence of human mammary epithelial cells overexpressing the Gab2 docking protein. J Biol Chem. 2006;281(1):626–37. 16253990 10.1074/jbc.M509567200

[25] Tan KW , Evrard M , Tham M , Hong M , Huang C , Kato M , et al. Tumor stroma and chemokines control T‐cell migration into melanoma following temozolomide treatment. Onco Targets Ther. 2015;4(2):e978709. 10.4161/2162402X.2014.978709 PMC4404877 25949877

[26] Hagihara K , Chan S , Zhang L , Oh DY , Wei XX , Simko J , et al. Neoadjuvant sipuleucel‐T induces both Th1 activation and immune regulation in localized prostate cancer. Onco Targets Ther. 2019;8(1):e1486953. 10.1080/2162402X.2018.1486953 PMC6287789 30546940

[27] Spanopoulou A , Gkretsi V . Growth differentiation factor 15 (GDF15) in cancer cell metastasis: from the cells to the patients. Clin Exp Metastasis. 2020;37(4):451–64. 32504264 10.1007/s10585-020-10041-3

[28] Benschop R , Wei T , Na S . Tumor necrosis factor receptor superfamily member 21: TNFR‐related death receptor‐6, DR6. Adv Exp Med Biol. 2009;647:186–94. 19760075 10.1007/978-0-387-89520-8_13

[29] Seth D , Shaw K , Jazayeri J , Leedman PJ . Complex post‐transcriptional regulation of EGF‐receptor expression by EGF and TGF‐alpha in human prostate cancer cells. Br J Cancer. 1999;80(5–6):657–69. 10360641 10.1038/sj.bjc.6690407 PMC2362295

[30] Hobor S , Van Emburgh BO , Crowley E , Misale S , Di Nicolantonio F , Bardelli A . TGFalpha and amphiregulin paracrine network promotes resistance to EGFR blockade in colorectal cancer cells. Clin Cancer Res. 2014;20(24):6429–38. 24916700 10.1158/1078-0432.CCR-14-0774

[31] Ha CT , Cheng CY , Zheng MY , Hsu TH , Miao CC , Lee CJ , et al. ID4 predicts poor prognosis and promotes BDNF‐mediated oncogenesis of colorectal cancer. Carcinogenesis. 2021;42(7):951–60. 33993270 10.1093/carcin/bgab037

[32] Shariat SF , Kattan MW , Traxel E , Andrews B , Zhu K , Wheeler TM , et al. Association of pre‐ and postoperative plasma levels of transforming growth factor beta(1) and interleukin 6 and its soluble receptor with prostate cancer progression. Clin Cancer Res. 2004;10(6):1992–9. 15041717 10.1158/1078-0432.ccr-0768-03

[33] Dopeso H , Jiao HK , Cuesta AM , Henze AT , Jurida L , Kracht M , et al. PHD3 controls lung cancer metastasis and resistance to EGFR inhibitors through TGFalpha. Cancer Res. 2018;78(7):1805–19. 29339541 10.1158/0008-5472.CAN-17-1346

[34] Tian M , Chen L , Ma L , Wang D , Shao B , Wu J , et al. Expression and prognostic significance of CCL11/CCR3 in glioblastoma. Oncotarget. 2016;7(22):32617–27. 27119233 10.18632/oncotarget.8958 PMC5078038

[35] Chen J , Zhang W , Wang Y , Zhao D , Wu M , Fan J , et al. The diacylglycerol kinase alpha (DGKalpha)/Akt/NF‐kappaB feedforward loop promotes esophageal squamous cell carcinoma (ESCC) progression via FAK‐dependent and FAK‐independent manner. Oncogene. 2019;38(14):2533–50. 30532074 10.1038/s41388-018-0604-6

[36] Ham IH , Oh HJ , Jin H , Bae CA , Jeon SM , Choi KS , et al. Targeting interleukin‐6 as a strategy to overcome stroma‐induced resistance to chemotherapy in gastric cancer. Mol Cancer. 2019;18(1):68. 30927911 10.1186/s12943-019-0972-8 PMC6441211

[37] Scanu A , Molnarfi N , Brandt KJ , Gruaz L , Dayer JM , Burger D . Stimulated T cells generate microparticles, which mimic cellular contact activation of human monocytes: differential regulation of pro‐ and anti‐inflammatory cytokine production by high‐density lipoproteins. J Leukoc Biol. 2008;83(4):921–7. 18223103 10.1189/jlb.0807551

[38] Nitta CF , Orlando RA . Crosstalk between immune cells and adipocytes requires both paracrine factors and cell contact to modify cytokine secretion. PLoS One. 2013;8(10):e77306. 24204798 10.1371/journal.pone.0077306 PMC3804580

[39] Chen YI , Chang CC , Hsu MF , Jeng YM , Tien YW , Chang MC , et al. Homophilic ATP1A1 binding induces activin a secretion to promote EMT of tumor cells and myofibroblast activation. Nat Commun. 2022;13(1):2945. 35618735 10.1038/s41467-022-30638-4 PMC9135720

[40] Wallner C , Drysch M , Becerikli M , Jaurich H , Wagner JM , Dittfeld S , et al. Interaction with the GDF8/11 pathway reveals treatment options for adenocarcinoma of the breast. Breast. 2018;37:134–41. 29156385 10.1016/j.breast.2017.11.010

[41] Simoni‐Nieves A , Gerardo‐Ramirez M , Pedraza‐Vazquez G , Chavez‐Rodriguez L , Bucio L , Souza V , et al. GDF11 implications in cancer biology and metabolism. Facts and Controversies Front Oncol. 2019;9:1039. 31681577 10.3389/fonc.2019.01039 PMC6803553

[42] Nogai H , Rosowski M , Grun J , Rietz A , Debus N , Schmidt G , et al. Follistatin antagonizes transforming growth factor‐beta3‐induced epithelial‐mesenchymal transition in vitro: implications for murine palatal development supported by microarray analysis. Differentiation. 2008;76(4):404–16. 18028449 10.1111/j.1432-0436.2007.00223.x

[43] Risbridger GP , Mellor SL , McPherson SJ , Schmitt JF . The contribution of inhibins and activins to malignant prostate disease. Mol Cell Endocrinol. 2001;180(1–2):149–53. 11451585 10.1016/s0303-7207(01)00497-x

[44] Gold E , Risbridger G . Activins and activin antagonists in the prostate and prostate cancer. Mol Cell Endocrinol. 2012;359(1–2):107–12. 21787836 10.1016/j.mce.2011.07.005

[45] Cambuli F , Foletto V , Alaimo A , De Felice D , Gandolfi F , Palumbieri MD , et al. Intra‐epithelial non‐canonical activin a signaling safeguards prostate progenitor quiescence. EMBO Rep. 2022;23(5):e54049. 35253958 10.15252/embr.202154049 PMC9066067

[46] Chen L , De Menna M , Groenewoud A , Thalmann GN , Kruithof‐de Julio M , Snaar‐Jagalska BE . A NF‐kB‐activin a signaling axis enhances prostate cancer metastasis. Oncogene. 2020;39(8):1634–51. 31740783 10.1038/s41388-019-1103-0

[47] Glister C , Kemp CF , Knight PG . Bone morphogenetic protein (BMP) ligands and receptors in bovine ovarian follicle cells: actions of BMP‐4, ‐6 and ‐7 on granulosa cells and differential modulation of Smad‐1 phosphorylation by follistatin. Reproduction. 2004;127(2):239–54. 15056790 10.1530/rep.1.00090

[48] van Schaik RH , Wierikx CD , Timmerman MA , Oomen MH , van Weerden WM , van der Kwast TH , et al. Variations in activin receptor, inhibin/activin subunit and follistatin mRNAs in human prostate tumour tissues. Br J Cancer. 2000;82(1):112–7. 10638976 10.1054/bjoc.1999.0886 PMC2363208

[49] Shi L , Resaul J , Owen S , Ye L , Jiang WG . Clinical and therapeutic implications of Follistatin in solid Tumours. Cancer Genomics Proteomics. 2016;13(6):425–35. 27807065 10.21873/cgp.20005 PMC5219916

[50] Thomas TZ , Wang H , Niclasen P , O'Bryan MK , Evans LW , Groome NP , et al. Expression and localization of activin subunits and follistatins in tissues from men with high grade prostate cancer. J Clin Endocrinol Metab. 1997;82(11):3851–8. 9360551 10.1210/jcem.82.11.4374

[51] McPherson SJ , Thomas TZ , Wang H , Gurusinghe CJ , Risbridger GP . Growth inhibitory response to activin A and B by human prostate tumour cell lines, LNCaP and DU145. J Endocrinol. 1997;154(3):535–45. 9379131 10.1677/joe.0.1540535

[52] Choi K , Jang HY , Ahn JM , Hwang SH , Chung JW , Choi YS , et al. The association of the serum levels of myostatin, follistatin, and interleukin‐6 with sarcopenia, and their impacts on survival in patients with hepatocellular carcinoma. Clin Mol Hepatol. 2020;26(4):492–505. 32646201 10.3350/cmh.2020.0005 PMC7641544

[53] Tomoda T , Nouso K , Miyahara K , Kobayashi S , Kinugasa H , Toyosawa J , et al. Prognotic impact of serum follistatin in patients with hepatocellular carcinoma. J Gastroenterol Hepatol. 2013;28(8):1391–6. 23432377 10.1111/jgh.12167

[54] Amedei A , Prisco D , MM DE . The use of cytokines and chemokines in the cancer immunotherapy. Recent Pat Anticancer Drug Discov. 2013;8(2):126–42. 22894642

[55] Sorrentino C , Ciummo SL , D'Antonio L , Fieni C , Lanuti P , Turdo A , et al. Interleukin‐30 feeds breast cancer stem cells via CXCL10 and IL23 autocrine loops and shapes immune contexture and host outcome. J Immunother Cancer. 2021;9(10):e002966. 34663639 10.1136/jitc-2021-002966 PMC8524378

[56] Begley LA , Kasina S , MacDonald J , Macoska JA . The inflammatory microenvironment of the aging prostate facilitates cellular proliferation and hypertrophy. Cytokine. 2008;43(2):194–9. 18572414 10.1016/j.cyto.2008.05.012 PMC2538565

[57] Lefort K , Ostano P , Mello‐Grand M , Calpini V , Scatolini M , Farsetti A , et al. Dual tumor suppressing and promoting function of Notch1 signaling in human prostate cancer. Oncotarget. 2016;7(30):48011–26. 27384993 10.18632/oncotarget.10333 PMC5216996

[58] Darash‐Yahana M , Gillespie JW , Hewitt SM , Chen YY , Maeda S , Stein I , et al. The chemokine CXCL16 and its receptor, CXCR6, as markers and promoters of inflammation‐associated cancers. PLoS One. 2009;4(8):e6695. 19690611 10.1371/journal.pone.0006695 PMC2723911

[59] Lin HM , Yeung N , Hastings JF , Croucher DR , Huynh K , Meikle TG , et al. Relationship between circulating lipids and cytokines in metastatic castration‐resistant prostate cancer. Cancers (Basel). 2021;13(19):4964. 34638448 10.3390/cancers13194964 PMC8508038

[60] Kapur N , Mir H , Sonpavde GP , Jain S , Bae S , Lillard JW Jr , et al. Prostate cancer cells hyper‐activate CXCR6 signaling by cleaving CXCL16 to overcome effect of docetaxel. Cancer Lett. 2019;454:1–13. 30974114 10.1016/j.canlet.2019.04.001 PMC7748218

[61] Wang J , Lu Y , Wang J , Koch AE , Zhang J , Taichman RS . CXCR6 induces prostate cancer progression by the AKT/mammalian target of rapamycin signaling pathway. Cancer Res. 2008;68(24):10367–76. 19074906 10.1158/0008-5472.CAN-08-2780 PMC2884407

