# Supplementary material for: Integrative characterisation of secreted factors involved in intercellular communication between prostate epithelial or cancer cells and fibroblasts
Source: Mol Oncol. 2023 Jan 27;17(3):469–86. doi: 10.1002/1878-0261.13376 (PMC9980303; doi:10.1002/1878-0261.13376)
Supplement: Supplementary file 1 — Fig. S1. Secretome of mono‐cultured prostate epithelial/cancer cells and patient‐derived fibroblasts. Fig. S2. Secretome of co‐cultured prostate epithelial/cancer cells with NPFs/CAFs. Fig. S3. Schematic of data visualisation by the X‐plot. Fig. S4. X‐plot approach to visualise how secretomes of co‐cultures of BPH‐1 with NPFs or CAFs differ from corresponding mono‐cultures. Fig. S5. X‐Plot highlighting proteins with differential expression between NPF and CAF co‐cultures. Fig. S6. Secretome comparison between co‐cultures of PC3 with NPFs or CAFs and corresponding mono‐cultures. Fig. S7. X‐Plot highlighting proteins with differential expression changes in PC3 co‐cultures with NPFs or CAFs. Fig. S8. Expression levels of secreted factors in primary and secondary cytokine/chemokine array screens. Fig. S9. A random cell migration assay identifies FST as a critical regulator in the co‐culture system. Fig. S10. FST knockdown in WPMY‐1 and PC3‐GFP cells by CRISPRi. Fig. S11. Stable knockdown of FST in both cell types by CRISPRi impairs migration of prostate cancer cells in co‐culture. Fig. S12. Human recombinant FST rescues the decreased migration of prostate cancer cells in co‐culture caused by stable FST knockdown. Fig. S13. Human recombinant FST rescues the impaired proliferation of prostate cancer cells in co‐culture mediated by stable FST knockdown. Fig. S14. FST expression and knockdown in different prostate epithelial/cancer cell lines. Fig. S15. FST regulates migration of additional prostate epithelial/cancer cell lines in co‐culture with fibroblasts. Fig. S16. Relationship of tumoural FST expression to disease‐free survival of patients with prostate cancer. Table S2. siRNA sequences used. Table S3. Summary of sgRNA sequences and primers. Table S4. Real‐time PCR primers. [file MOL2-17-469-s001.pdf]

Integrative characterisation of secreted factors involved in intercellular communication between prostate epithelial or cancer cells and fibroblasts

Yunjian Wu, Kimberley C. Clark, Birunthi Niranjan, Anderly C. Chüeh, Lisa G. Horvath, Renea A. Taylor and Roger J. Daly.

Supplementary Figures

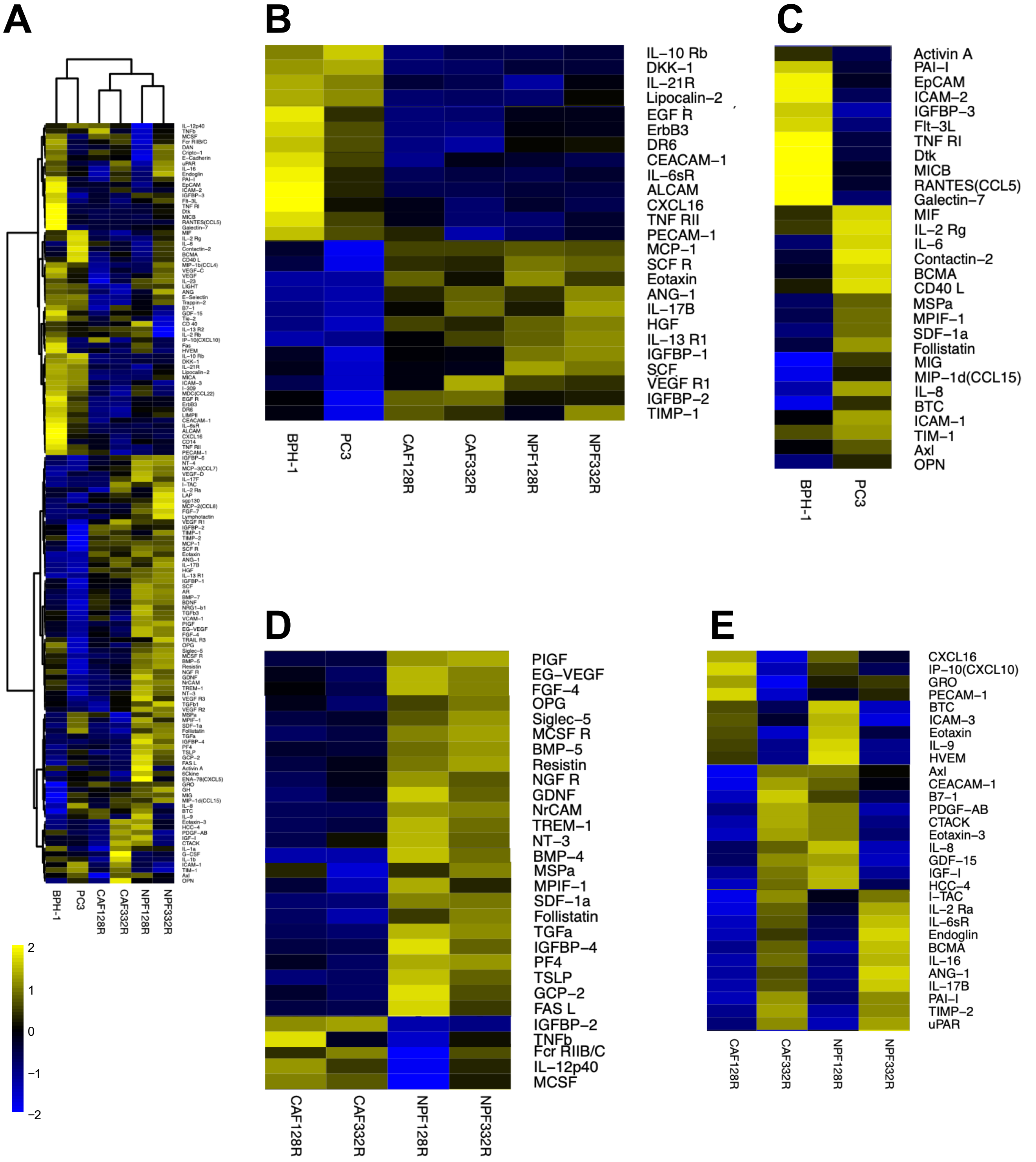

Fig. S1. Secretome of mono-cultured prostate epithelial/cancer cells and patient-derived fibroblasts. **A**, Secretion patterns of each mono-cultured cell line (BPH-1, PC3 and NPF/CAF from patients 128R and 332R). Log 10 data were z-score transformed as shown on the scale. **B**, Examples of different secretion patterns between prostate epithelial/cancer cells and NPF/CAF conditioned media. **C**, Examples of different secretion patterns between BPH-1 and PC3 conditioned media. **D**, Examples of different secretion patterns between NPF and CAF conditioned media. **E**, Examples of inconsistent secretion patterns across different patient samples. The primary screen was undertaken with one biological replicate. CAF, cancer-associated fibroblast; NPF, non-malignant prostate fibroblast.

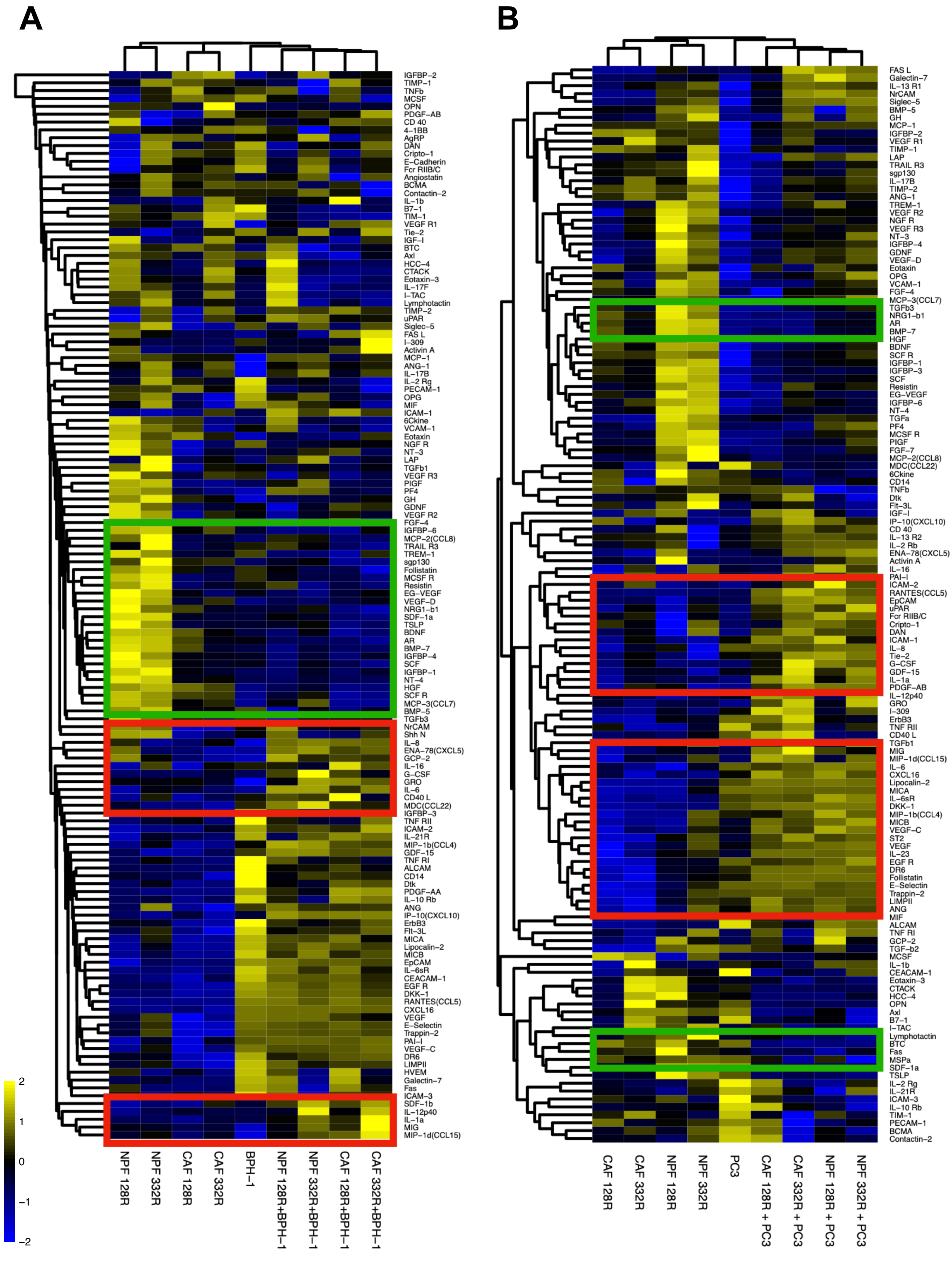
A
IGFBP-2
TIMP-1
TNFb
MCSF
OPN
PDGF-AB
CD 40
4-1BB
AgRP
DAN
Cripto-1
E-Cadherin
Fcr RIIB/C
Angiostatin
BCMA
Contactin-2
IL-1b
B7-1
TIM-1
VEGF R1
Tie-2
IGF-I
BTC
Axl
HCC-4
CTACK
Eotaxin-3
IL-17F
I-TAC
Lymphotactin
TIMP-2
uPAR
Siglec-5
FAS L
I-309
Activin A
MCP-1
ANG-1
IL-17B
IL-2 Rg
PECAM-1
OPG
MIF
ICAM-1
6Ckine
VCAM-1
Eotaxin
NGF R
NT-3
LAP
TGFb1
VEGF R3
PIGF
PF4
GH
GDNF
VEGF R2
FGF-4
IGFBP-6
MCP-2(CCL8)
TRAIL R3
TREM-1
sgp130
Follistatin
MCSF R
Resistin
EG-VEGF
VEGF-D
NRG1-b1
SDF-1a
TSLP
BDNF
AR
BMP-7
IGFBP-4
SCF
IGFBP-1
NT-4
HGF
SCF R
MCP-3(CCL7)
BMP-5
TGFb3
NrCAM
Shh N
IL-8
ENA-78(CXCL5)
GCP-2
IL-16
G-CSF
GRO
IL-6
CD40 L
MDC(CCL22)
IGFBP-3
TNF RII
ICAM-2
IL-21R
MIP-1b(CCL4)
GDF-15
TNF RI
ALCAM
CD14
Dtk
PDGF-AA
IL-10 Rb
ANG
IP-10(CXCL10)
ErbB3
Flt-3L
MICA
Lipocalin-2
MICB
EpCAM
IL-6sR
CEACAM-1
EGF R
DKK-1
RANTES(CCL5)
CXCL16
VEGF
E-Selectin
Trappin-2
PAI-I
VEGF-C
DR6
LIMPII
HVEM
Galectin-7
Fas
ICAM-3
SDF-1b
IL-12p40
IL-1a
MIG
MIP-1d(CCL15)
2
1
0
−1
−2
NPF 128R
NPF 332R
CAF 128R
CAF 332R
BPH-1
NPF 128R+BPH-1
NPF 332R+BPH-1
CAF 128R+BPH-1
CAF 332R+BPH-1
B
FAS L
Galectin-7
IL-13 R1
NrCAM
Siglec-5
BMP-5
GH
MCP-1
IGFBP-2
VEGF R1
TIMP-1
LAP
TRAIL R3
sgp130
IL-17B
TIMP-2
ANG-1
TREM-1
VEGF R2
NGF R
VEGF R3
NT-3
IGFBP-4
GDNF
VEGF-D
Eotaxin
OPG
VCAM-1
FGF-4
MCP-3(CCL7)
TGFb3
NRG1-b1
AR
BMP-7
HGF
BDNF
SCF R
IGFBP-1
IGFBP-3
SCF
Resistin
EG-VEGF
IGFBP-6
NT-4
TGFa
PF4
MCSF R
PIGF
FGF-7
MCP-2(CCL8)
MDC(CCL22)
6Ckine
CD14
TNFb
Dtk
Flt-3L
IGF-I
IP-10(CXCL10)
CD 40
IL-13 R2
IL-2 Rb
ENA-78(CXCL5)
Activin A
IL-16
PAI-I
ICAM-2
RANTES(CCL5)
EpCAM
uPAR
Fcr RIIB/C
Cripto-1
DAN
ICAM-1
IL-8
Tie-2
G-CSF
GDF-15
IL-1a
PDGF-AB
IL-12p40
GRO
I-309
ErbB3
TNF RII
CD40 L
TGFb1
MIG
MIP-1d(CCL15)
IL-6
CXCL16
Lipocalin-2
MICA
IL-6sR
DKK-1
MIP-1b(CCL4)
MICB
VEGF-C
ST2
VEGF
IL-23
EGF R
DR6
Follistatin
E-Selectin
Trappin-2
LIMPII
ANG
MIF
ALCAM
TNF RI
GCP-2
TGF-b2
MCSF
IL-1b
CEACAM-1
Eotaxin-3
CTACK
HCC-4
OPN
Axl
B7-1
I-TAC
Lymphotactin
BTC
Fas
MSPa
SDF-1a
TSLP
IL-2 Rg
IL-21R
ICAM-3
IL-10 Rb
TIM-1
PECAM-1
BCMA
Contactin-2
CAF 128R
CAF 332R
NPF 128R
NPF 332R
PC3
CAF 128R + PC3
CAF 332R + PC3
NPF 128R + PC3
NPF 332R + PC3

Fig. S2. Secretome of co-cultured prostate epithelial/cancer cells with NPFs/CAFs. Unsupervised hierarchical clustering of secreted factors from (**A**) BPH-1 co-cultured with fibroblasts and (**B**) PC3 co-cultured with fibroblasts reveals culture condition-specific clusters (red boxes highlight secreted factors showing up-regulation in co-culture versus mono-culture, and green boxes highlight secreted factors showing down-regulation in co-culture versus mono-culture). Log 10 data were z-score transformed as shown on the scale. The primary screen was undertaken with one biological replicate. CAF, cancer-associated fibroblast; NPF, non-malignant prostate fibroblast.

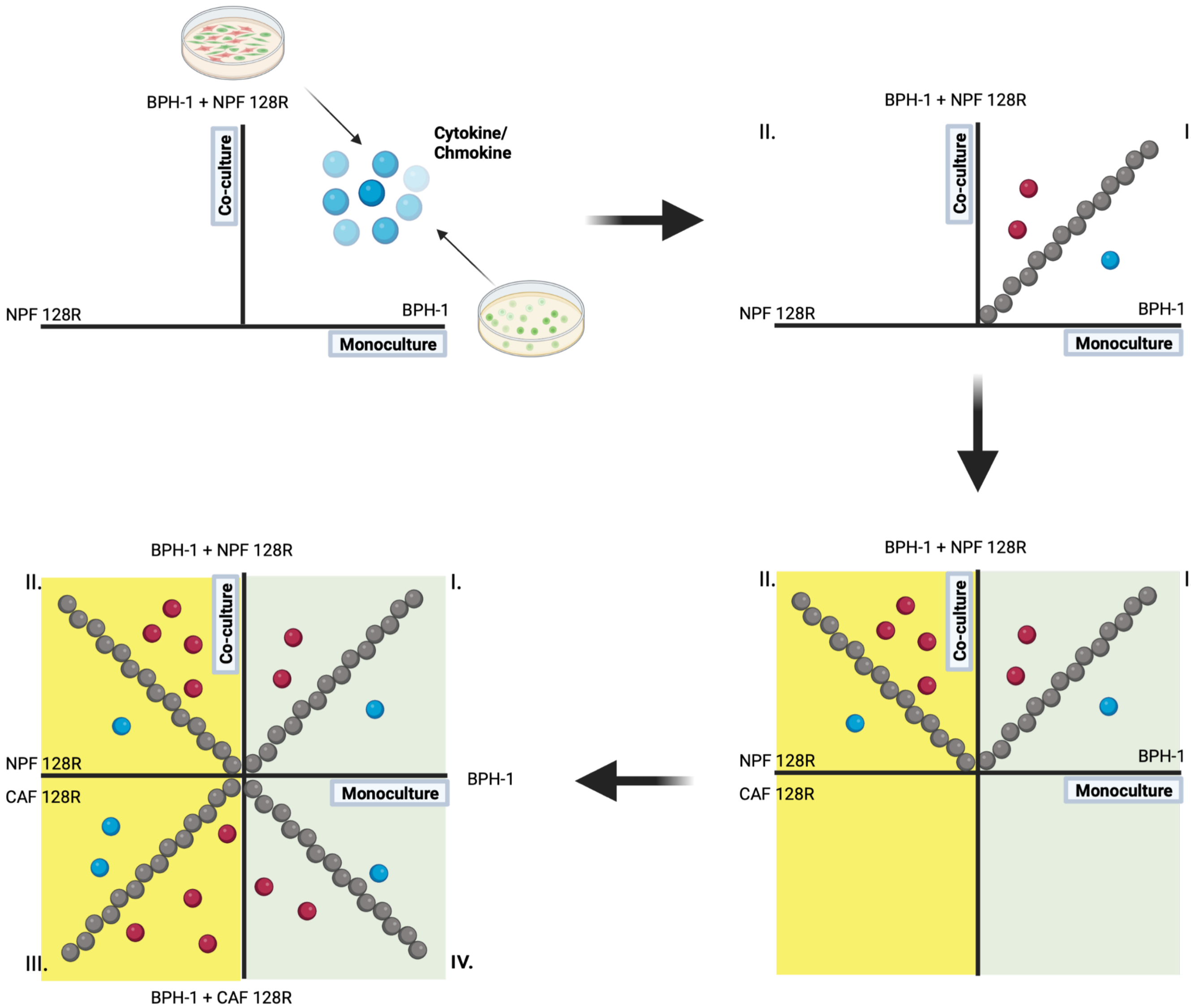

Fig. S3. Schematic of data visualisation by the X-plot. Approach utilises BPH-1 co-culture with NPF/CAF 128R as an example. The x-axis indicates expression of secreted factors in mono-culture, the y-axis, in co-culture. The pale green background highlights BPH-1 cytokine/chemokine secretion, yellow background, fibroblast secretion. Secreted factors closer to the y-axis represent an increase of expression

in co-culture while factors closer to the x-axis represent a decrease in expression (Created with BioRender software). CAF, cancer-associated fibroblast; NPF, non-malignant prostate fibroblast.

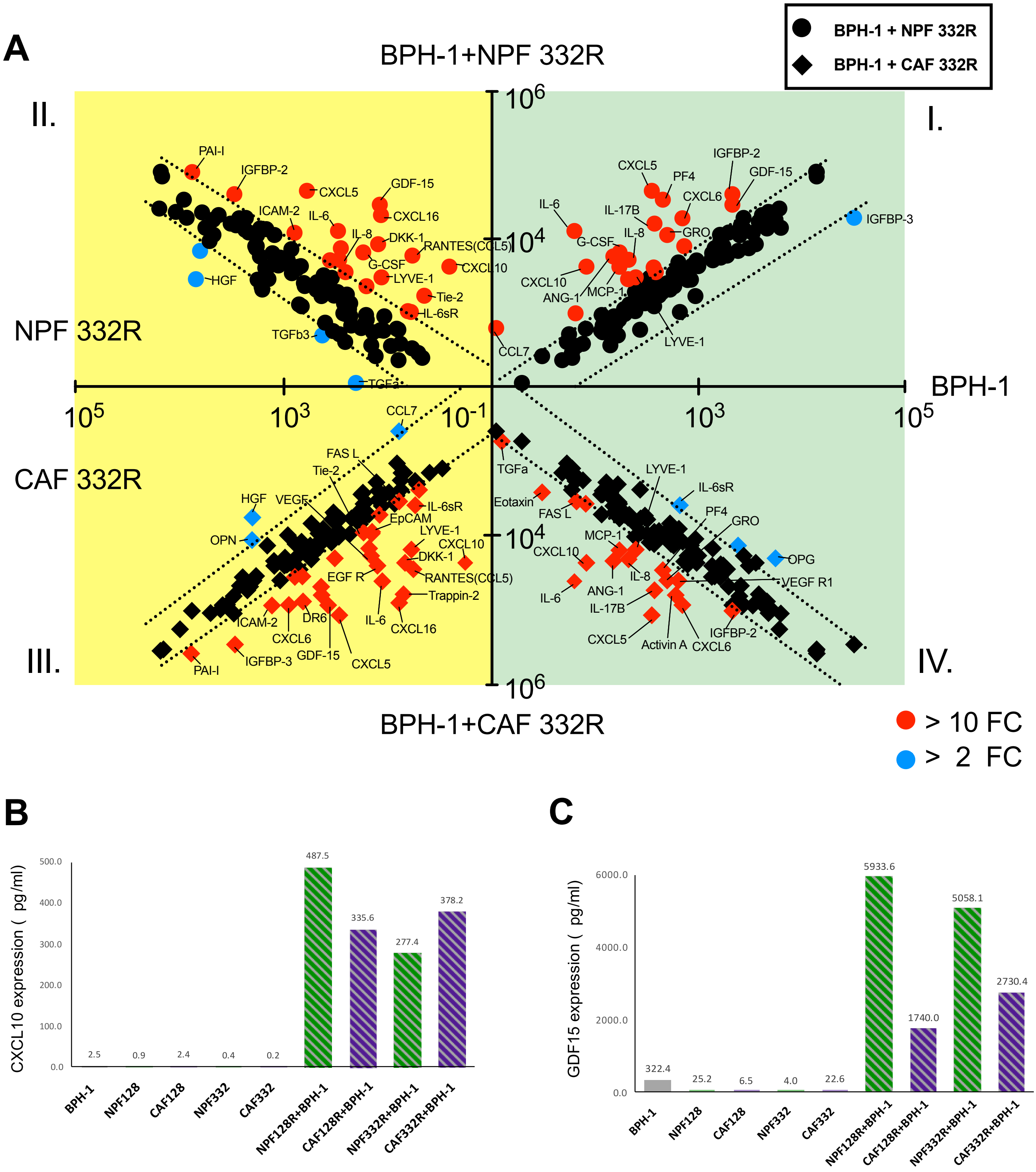

Fig. S4. X-plot approach to visualise how secretomes of co-cultures of BPH-1 with NPFs or CAFs differ from corresponding mono-cultures. **A**, X plot for BPH-1+NPF/CAF 332R cultures where up-regulated cytokine/chemokines (red dots) were selected using a stringent cut-off value of FC>10 (protein level in co-culture/corresponding mono-culture), and decreased cytokine/chemokine (blue dots) was selected by a cut-off value of > 2 FC (protein level in mono-culture/corresponding co-culture). Green background: BPH-1 cytokine/chemokine secretion, yellow background: fibroblast cytokine/chemokine secretion. **B** and **C**, Expression levels of CXCL10 (**B**) and GDF15 (**C**) in co-cultures and mono-cultures. The primary screen was undertaken with one biological replicate. FC, fold change; CAF, cancer-associated fibroblast; NPF, non-malignant prostate fibroblast.

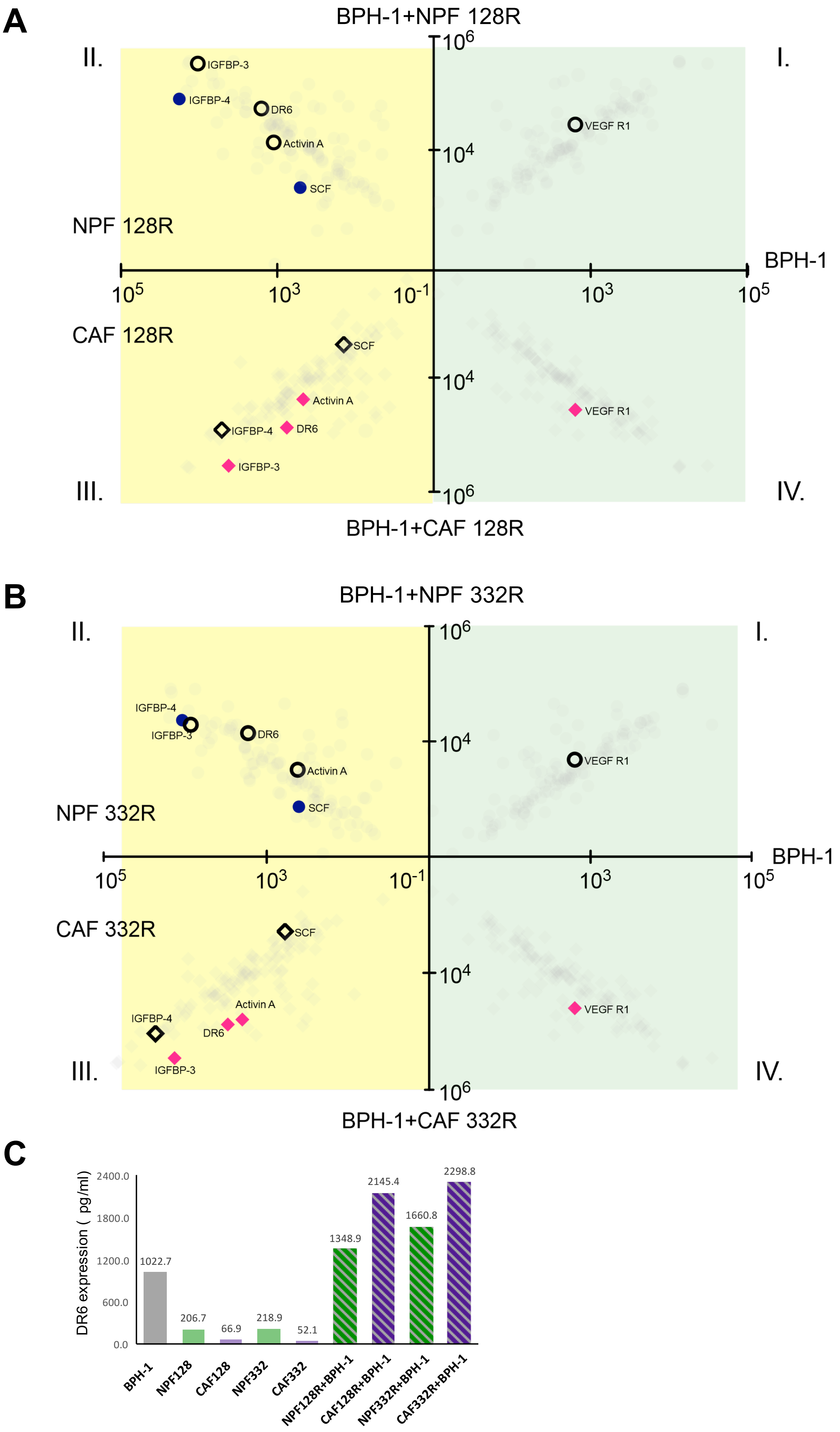
A
BPH-1+NPF 128R
II.
I.
IGFBP-3
IGFBP-4
DR6
Activin A
SCF
VEGF R1
NPF 128R
BPH-1
CAF 128R
SCF
Activin A
IGFBP-4
DR6
IGFBP-3
VEGF R1
III.
IV.
BPH-1+CAF 128R
B
BPH-1+NPF 332R
II.
I.
IGFBP-4
IGFBP-3
DR6
Activin A
SCF
VEGF R1
NPF 332R
BPH-1
CAF 332R
SCF
Activin A
IGFBP-4
DR6
IGFBP-3
VEGF R1
III.
IV.
BPH-1+CAF 332R
C
DR6 expression (pg/ml)
2400.0
1800.0
1200.0
600.0
0.0
1022.7
206.7
66.9
218.9
52.1
1348.9
2145.4
1660.8
2298.8
BPH-1
NPF128
CAF128
NPF332
CAF332
NPF128R+BPH-1
CAF128R+BPH-1
NPF332R+BPH-1
CAF332R+BPH-1

Fig. S5. X-Plot highlighting proteins with differential expression between NPF and CAF co-cultures. **A** and **B**, X-plots highlighting proteins with differential expression between co-cultures with NPFs or CAFs for BPH-1+NPF/CAF 128R co-culture (**A**) and BPH-1+NPF/CAF 332R co-culture (**B**). Up-regulated cytokine/chemokines (pink dots) were selected using a stringent cut-off value of FC>10 (protein level in co-culture/corresponding mono-culture), and decreased cytokine/chemokine (blue dots) was selected by a cut-off value of > 2 FC (protein level in mono-culture/corresponding co-culture). Hollow dots represent proteins with no change in co-culture compared to the corresponding mono-cultures. Pale green background highlights BPH-1 cytokine/chemokine secretion, yellow background, fibroblast secretion. **C,** DR6 expression level in co-cultures and mono-cultures. The primary screen was undertaken with one biological replicate. FC, fold change; CAF, cancer-associated fibroblast; NPF, non-malignant prostate fibroblast.

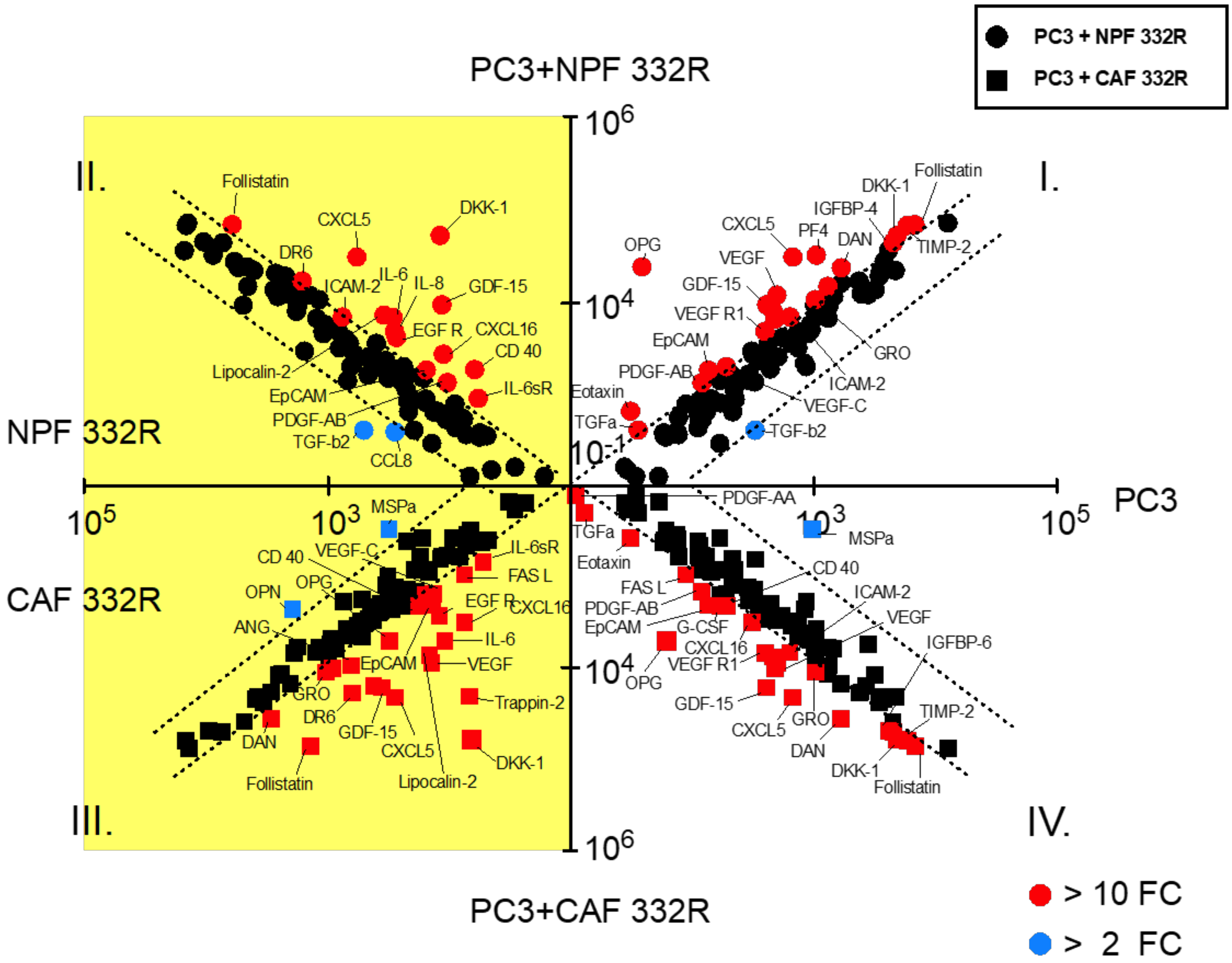

Fig. S6. Secretome comparison between co-cultures of PC3 with NPFs or CAFs and corresponding mono-cultures. A. X plot for PC3+NPF/CAF332R co-culture where up-regulated cytokine/chemokines (red dots) were selected using a stringent cut-off value of FC>10 (protein level in co-culture/corresponding mono-culture), and decreased cytokine/chemokine (blue dots) was selected by a cut-off value of > 2 FC (protein level in mono-culture/corresponding co-culture). White background indicates PC3 cytokine/chemokine secretion, yellow background, fibroblast secretion. The primary screen was undertaken with one biological replicate. FC, fold change; CAF, cancer-associated fibroblast; NPF, non-malignant prostate fibroblast.

A

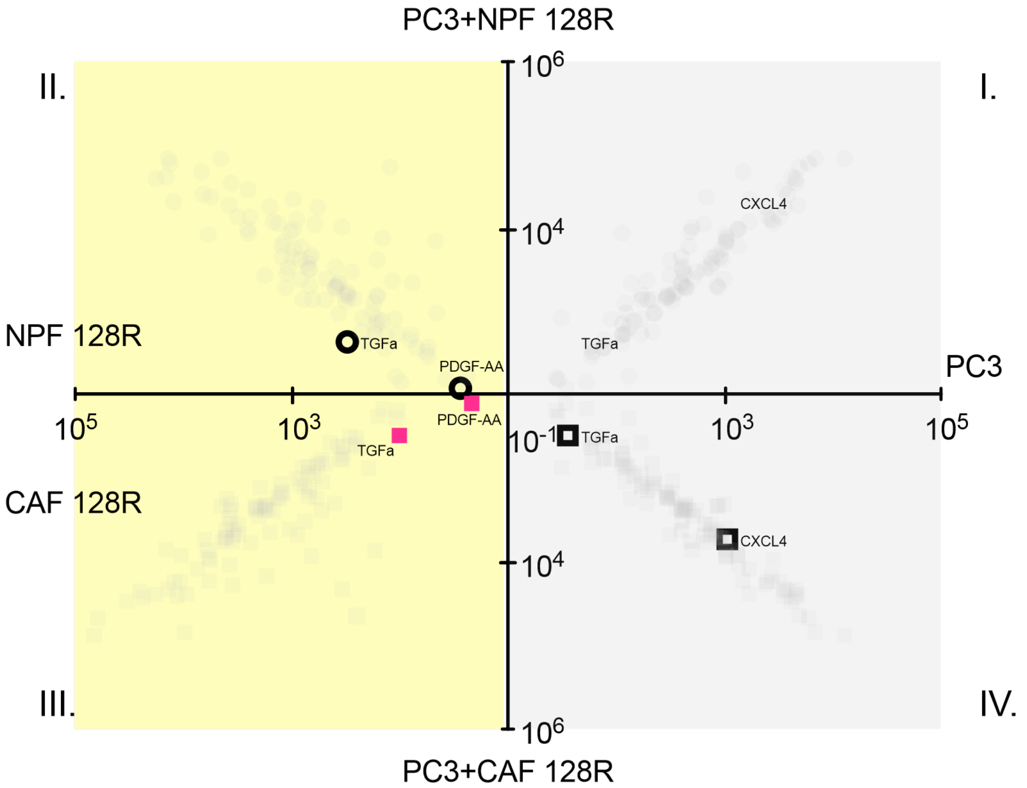
PC3+NPF 128R
II.
I.
III.
IV.
NPF 128R
CAF 128R
PC3
PC3+CAF 128R
10^6
10^4
10^5
10^3
10^-1
TGFa
PDGF-AA
CXCL4

B

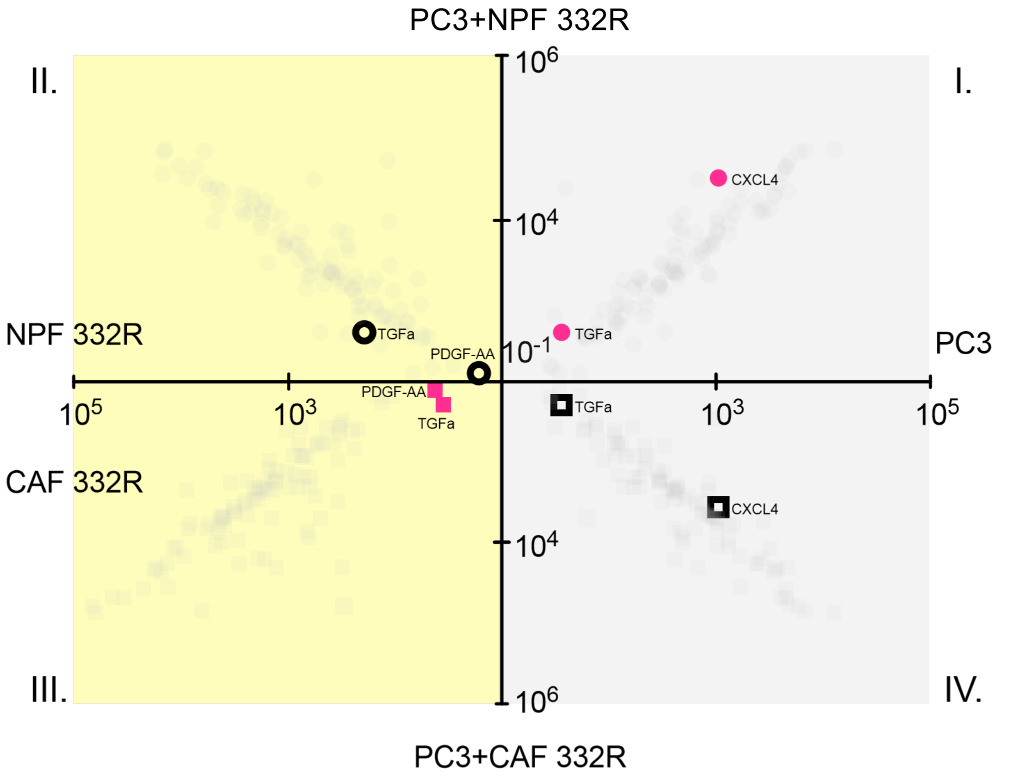
PC3+NPF 332R
II.
I.
III.
IV.
NPF 332R
CAF 332R
PC3
PC3+CAF 332R
10^6
10^4
10^5
10^3
10^-1
TGFa
PDGF-AA
CXCL4

C

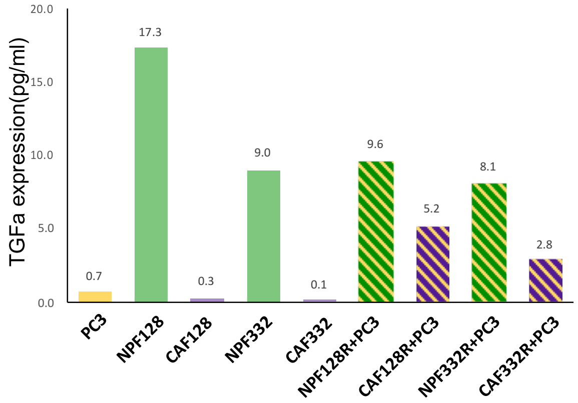
TGFa expression(pg/ml)
20.0
15.0
10.0
5.0
0.0
0.7
17.3
0.3
9.0
0.1
9.6
5.2
8.1
2.8
PC3
NPF128
CAF128
NPF332
CAF332
NPF128R+PC3
CAF128R+PC3
NPF332R+PC3
CAF332R+PC3

Fig. S7. X-Plot highlighting proteins with differential expression changes in PC3 co-cultures with NPFs or CAFs. **A** and **B**, X-plots highlighting proteins with differential regulation in co-cultures with NPFs or CAFs in PC3+NPF/CAF 128R co-culture (**A**) and PC3+NPF/CAF 332R co-culture (**B**). Up-regulated cytokine/chemokines (pink dots) were selected using a stringent cut-off value of FC>10 (protein level in co-culture/corresponding mono-culture). Hollow dots/boxes indicate proteins with no change in the co-culture compared to the corresponding mono-cultures. Grey background highlights PC3 cytokine/chemokine secretion, yellow background, fibroblast secretion. **C,** TGFα expression level in co-cultures and mono-cultures. The primary screen was undertaken with one biological replicate. FC, fold change; CAF, cancer-associated fibroblast; NPF, non-malignant prostate fibroblast.

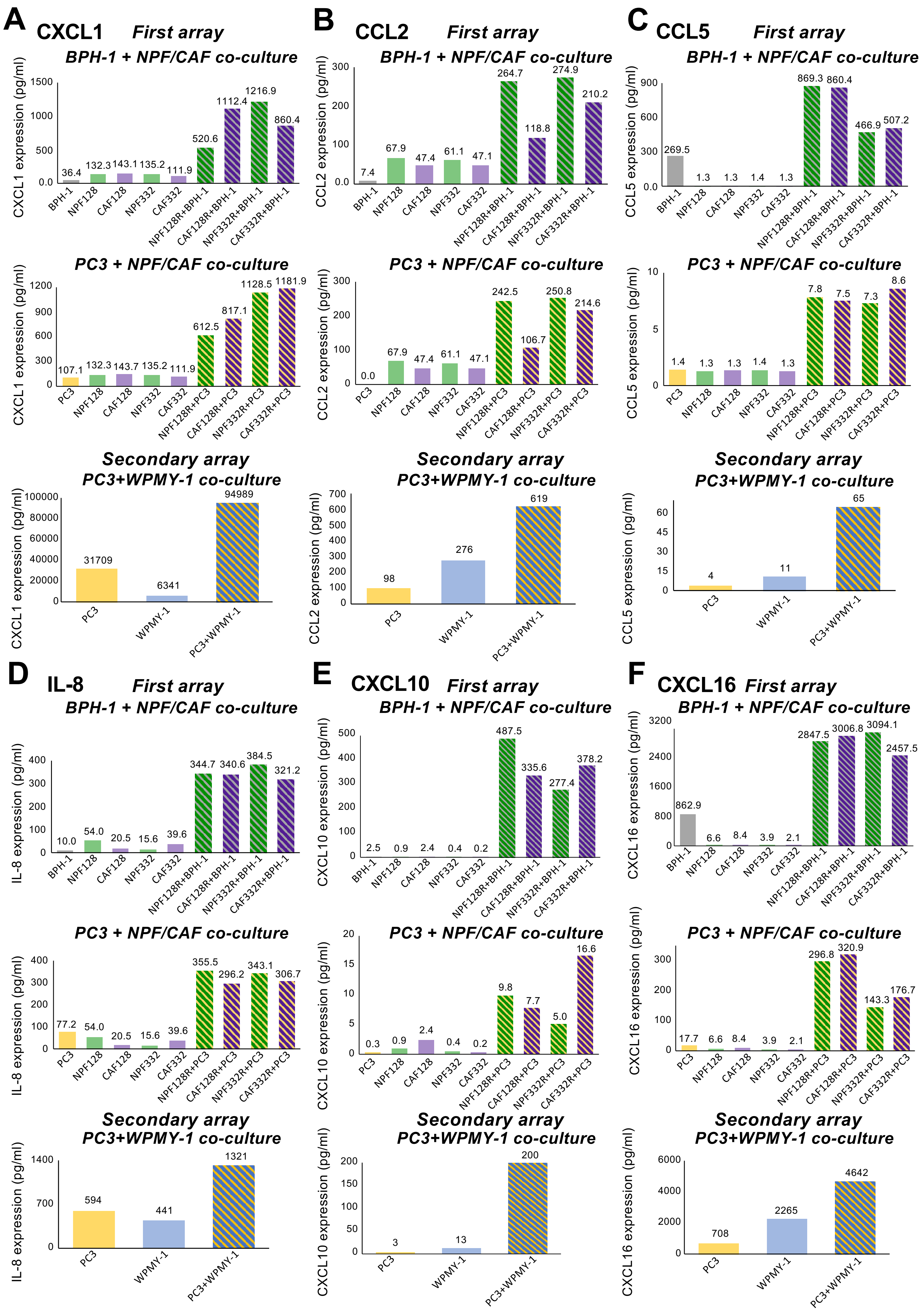
A
CXCL1 First array
BPH-1 + NPF/CAF co-culture
CXCL1 expression (pg/ml)
0.0
500
1000
1500
36.4
132.3
143.1
135.2
111.9
520.6
1112.4
1216.9
860.4
BPH-1
NPF128
CAF128
NPF332
CAF332
NPF128R+BPH-1
CAF128R+BPH-1
NPF332R+BPH-1
CAF332R+BPH-1
PC3 + NPF/CAF co-culture
CXCL1 expression (pg/ml)
0
300
600
900
1200
107.1
132.3
143.7
135.2
111.9
612.5
817.1
1128.5
1181.9
PC3
NPF128
CAF128
NPF332
CAF332
NPF128R+PC3
CAF128R+PC3
NPF332R+PC3
CAF332R+PC3
Secondary array
PC3+WPMY-1 co-culture
CXCL1 expression (pg/ml)
0
20000
40000
60000
80000
100000
31709
6341
94989
PC3
WPMY-1
PC3+WPMY-1
B
CCL2 First array
BPH-1 + NPF/CAF co-culture
CCL2 expression (pg/ml)
0.0
100
200
300
7.4
67.9
47.4
61.1
47.1
264.7
118.8
274.9
210.2
BPH-1
NPF128
CAF128
NPF332
CAF332
NPF128R+BPH-1
CAF128R+BPH-1
NPF332R+BPH-1
CAF332R+BPH-1
PC3 + NPF/CAF co-culture
CCL2 expression (pg/ml)
0
100
200
300
0.0
67.9
47.4
61.1
47.1
242.5
106.7
250.8
214.6
PC3
NPF128
CAF128
NPF332
CAF332
NPF128R+PC3
CAF128R+PC3
NPF332R+PC3
CAF332R+PC3
Secondary array
PC3+WPMY-1 co-culture
CCL2 expression (pg/ml)
0
100
200
300
400
500
600
700
98
276
619
PC3
WPMY-1
PC3+WPMY-1
C
CCL5 First array
BPH-1 + NPF/CAF co-culture
CCL5 expression (pg/ml)
0.0
300
600
900
269.5
1.3
1.3
1.4
1.3
869.3
860.4
466.9
507.2
BPH-1
NPF128
CAF128
NPF332
CAF332
NPF128R+BPH-1
CAF128R+BPH-1
NPF332R+BPH-1
CAF332R+BPH-1
PC3 + NPF/CAF co-culture
CCL5 expression (pg/ml)
0
5
10
1.4
1.3
1.3
1.4
1.3
7.8
7.5
7.3
8.6
PC3
NPF128
CAF128
NPF332
CAF332
NPF128R+PC3
CAF128R+PC3
NPF332R+PC3
CAF332R+PC3
Secondary array
PC3+WPMY-1 co-culture
CCL5 expression (pg/ml)
0
15
30
45
60
4
11
65
PC3
WPMY-1
PC3+WPMY-1
D
IL-8 First array
BPH-1 + NPF/CAF co-culture
IL-8 expression (pg/ml)
0
100
200
300
400
10.0
54.0
20.5
15.6
39.6
344.7
340.6
384.5
321.2
BPH-1
NPF128
CAF128
NPF332
CAF332
NPF128R+BPH-1
CAF128R+BPH-1
NPF332R+BPH-1
CAF332R+BPH-1
PC3 + NPF/CAF co-culture
IL-8 expression (pg/ml)
0
100
200
300
400
77.2
54.0
20.5
15.6
39.6
355.5
296.2
343.1
306.7
PC3
NPF128
CAF128
NPF332
CAF332
NPF128R+PC3
CAF128R+PC3
NPF332R+PC3
CAF332R+PC3
Secondary array
PC3+WPMY-1 co-culture
IL-8 expression (pg/ml)
0
700
1400
594
441
1321
PC3
WPMY-1
PC3+WPMY-1
E
CXCL10 First array
BPH-1 + NPF/CAF co-culture
CXCL10 expression (pg/ml)
0
100
200
300
400
500
2.5
0.9
2.4
0.4
0.2
487.5
335.6
277.4
378.2
BPH-1
NPF128
CAF128
NPF332
CAF332
NPF128R+BPH-1
CAF128R+BPH-1
NPF332R+BPH-1
CAF332R+BPH-1
PC3 + NPF/CAF co-culture
CXCL10 expression (pg/ml)
0
5
10
15
20
0.3
0.9
2.4
0.4
0.2
9.8
7.7
5.0
16.6
PC3
NPF128
CAF128
NPF332
CAF332
NPF128R+PC3
CAF128R+PC3
NPF332R+PC3
CAF332R+PC3
Secondary array
PC3+WPMY-1 co-culture
CXCL10 expression (pg/ml)
0
50
100
150
200
3
13
200
PC3
WPMY-1
PC3+WPMY-1
F
CXCL16 First array
BPH-1 + NPF/CAF co-culture
CXCL16 expression (pg/ml)
0
800
1600
2400
3200
862.9
6.6
8.4
3.9
2.1
2847.5
3006.8
3094.1
2457.5
BPH-1
NPF128
CAF128
NPF332
CAF332
NPF128R+BPH-1
CAF128R+BPH-1
NPF332R+BPH-1
CAF332R+BPH-1
PC3 + NPF/CAF co-culture
CXCL16 expression (pg/ml)
0
100
200
300
17.7
6.6
8.4
3.9
2.1
296.8
320.9
143.3
176.7
PC3
NPF128
CAF128
NPF332
CAF332
NPF128R+PC3
CAF128R+PC3
NPF332R+PC3
CAF332R+PC3
Secondary array
PC3+WPMY-1 co-culture
CXCL16 expression (pg/ml)
0
2000
4000
6000
708
2265
4642
PC3
WPMY-1
PC3+WPMY-1

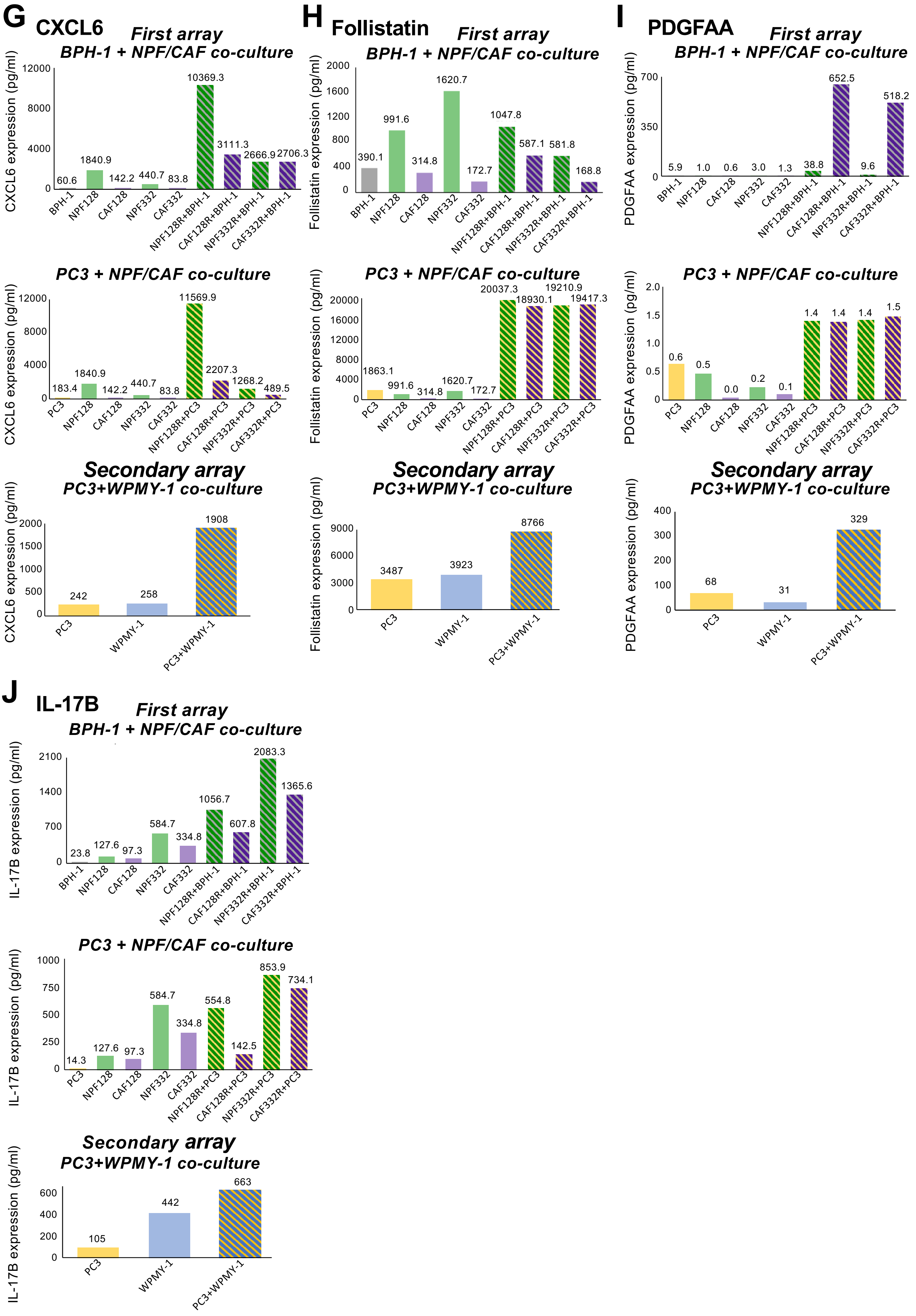

G
CXCL6
First array
BPH-1 + NPF/CAF co-culture
CXCL6 expression (pg/ml)
12000
8000
4000
0
60.6
1840.9
142.2
440.7
83.8
10369.3
3111.3
2666.9
2706.3
BPH-1
NPF128
CAF128
NPF332
CAF332
NPF128R+BPH-1
CAF128R+BPH-1
NPF332R+BPH-1
CAF332R+BPH-1
PC3 + NPF/CAF co-culture
CXCL6 expression (pg/ml)
12000
8000
4000
0
183.4
1840.9
142.2
440.7
83.8
11569.9
2207.3
1268.2
489.5
PC3
NPF128
CAF128
NPF332
CAF332
NPF128R+PC3
CAF128R+PC3
NPF332R+PC3
CAF332R+PC3
Secondary array
PC3+WPMY-1 co-culture
CXCL6 expression (pg/ml)
2000
1500
1000
500
0
242
258
1908
PC3
WPMY-1
PC3+WPMY-1
H
Follistatin
First array
BPH-1 + NPF/CAF co-culture
Follistatin expression (pg/ml)
2000
1600
1200
800
400
0
390.1
991.6
314.8
1620.7
172.7
1047.8
587.1
581.8
168.8
BPH-1
NPF128
CAF128
NPF332
CAF332
NPF128R+BPH-1
CAF128R+BPH-1
NPF332R+BPH-1
CAF332R+BPH-1
PC3 + NPF/CAF co-culture
Follistatin expression (pg/ml)
20000
16000
12000
8000
4000
0
1863.1
991.6
314.8
1620.7
172.7
20037.3
18930.1
19210.9
19417.3
PC3
NPF128
CAF128
NPF332
CAF332
NPF128R+PC3
CAF128R+PC3
NPF332R+PC3
CAF332R+PC3
Secondary array
PC3+WPMY-1 co-culture
Follistatin expression (pg/ml)
9000
6000
3000
0
3487
3923
8766
PC3
WPMY-1
PC3+WPMY-1
I
PDGFAA
First array
BPH-1 + NPF/CAF co-culture
PDGFAA expression (pg/ml)
700
350
0
5.9
1.0
0.6
3.0
1.3
38.8
652.5
9.6
518.2
BPH-1
NPF128
CAF128
NPF332
CAF332
NPF128R+BPH-1
CAF128R+BPH-1
NPF332R+BPH-1
CAF332R+BPH-1
PC3 + NPF/CAF co-culture
PDGFAA expression (pg/ml)
2.0
1.5
1.0
0.5
0
0.6
0.5
0.0
0.2
0.1
1.4
1.4
1.4
1.5
PC3
NPF128
CAF128
NPF332
CAF332
NPF128R+PC3
CAF128R+PC3
NPF332R+PC3
CAF332R+PC3
Secondary array
PC3+WPMY-1 co-culture
PDGFAA expression (pg/ml)
400
300
200
100
0
68
31
329
PC3
WPMY-1
PC3+WPMY-1
J
IL-17B
First array
BPH-1 + NPF/CAF co-culture
IL-17B expression (pg/ml)
2100
1400
700
0
23.8
127.6
97.3
584.7
334.8
1056.7
607.8
2083.3
1365.6
BPH-1
NPF128
CAF128
NPF332
CAF332
NPF128R+BPH-1
CAF128R+BPH-1
NPF332R+BPH-1
CAF332R+BPH-1
PC3 + NPF/CAF co-culture
IL-17B expression (pg/ml)
1000
750
500
250
0
14.3
127.6
97.3
584.7
334.8
554.8
142.5
853.9
734.1
PC3
NPF128
CAF128
NPF332
CAF332
NPF128R+PC3
CAF128R+PC3
NPF332R+PC3
CAF332R+PC3
Secondary array
PC3+WPMY-1 co-culture
IL-17B expression (pg/ml)
600
400
200
0
105
442
663
PC3
WPMY-1
PC3+WPMY-1

Fig. S8. Expression levels of secreted factors in primary and secondary cytokine/chemokine array screens. A-J, Array data for individual factors in the primary screen using primary patient-matched fibroblasts and secondary screen using WPMY-1 fibroblasts. The primary or secondary screen was undertaken with one biological replicate. CAF, cancer-associated fibroblast; NPF, non-malignant prostate fibroblast.

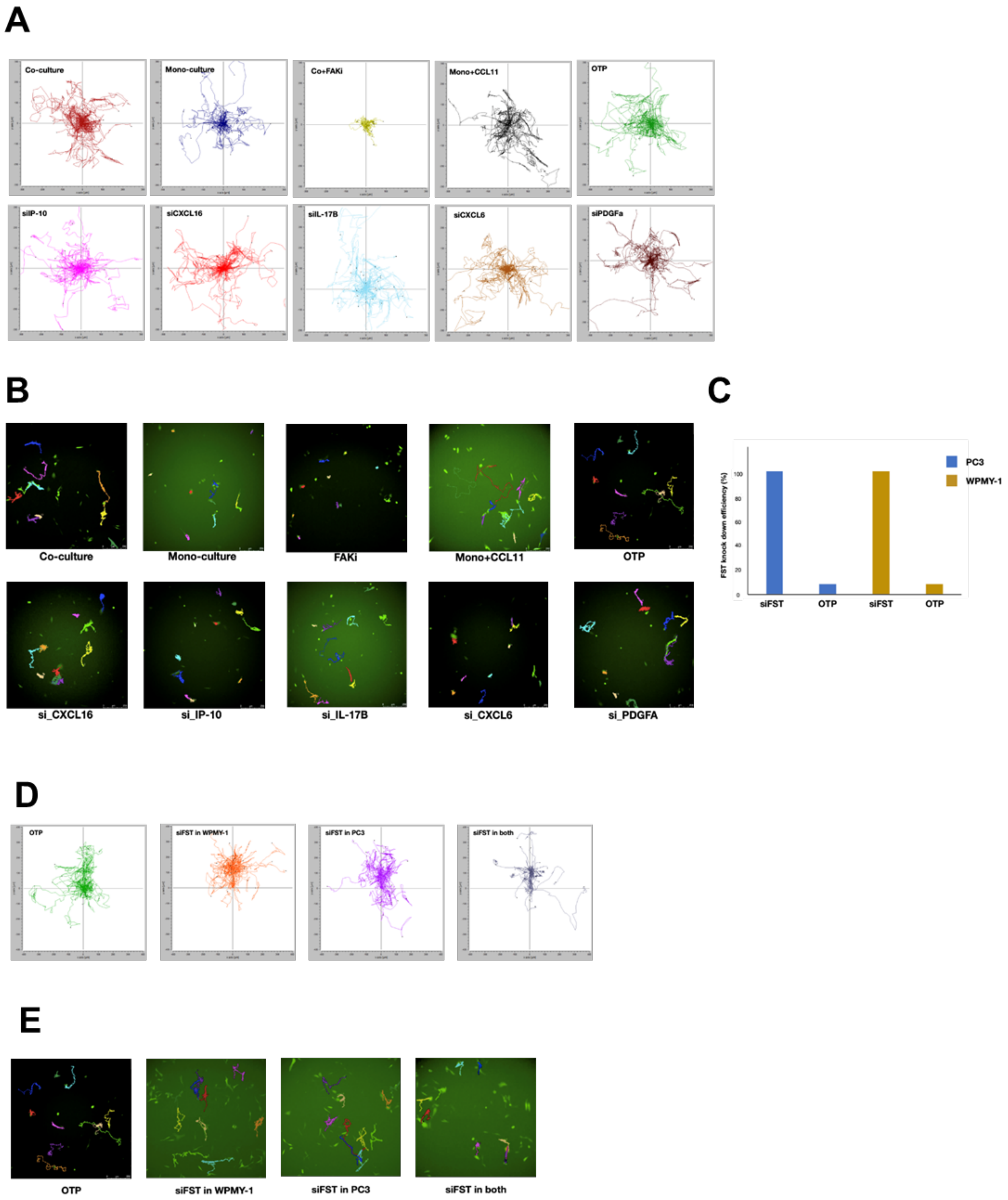
A
Co-culture
Mono-culture
Co+FAKi
Mono+CCL11
OTP
siIP-10
siCXCL16
siIL-17B
siCXCL6
siPDGFa
B
Co-culture
Mono-culture
FAKi
Mono+CCL11
OTP
si_CXCL16
si_IP-10
si_IL-17B
si_CXCL6
si_PDGFA
C
FST knock down efficiency (%)
PC3
WPMY-1
siFST
OTP
siFST
OTP
D
OTP
siFST in WPMY-1
siFST in PC3
siFST in both
E
OTP
siFST in WPMY-1
siFST in PC3
siFST in both

Fig. S9. A random cell migration assay identifies FST as a critical regulator in the co-culture system. The targets were knocked down by transfection with corresponding siRNA with ON-TARGETplus (OTP) as the negative control. **A**, Trajectories of PC3-GFP cells in the co-culture system when different targets were knocked down in both PC3-GFP and WPMY-1 cells, and CCL11 and FAKi were used as controls (representative of n=3). **B**, Examples of dragon tails display showing single PC3-GFP cell migration tracks in which temporal changes in cell location are indicated as coloured lines (last frames of the time-lapse movies). **C**, FST Knockdown efficiency in either PC3 or WPMY-1 cells was confirmed by RT-PCR (n=1). **D**, Trajectories of PC3-GFP cells in the co-culture system when FST was knocked down in either PC3-GFP or WPMY-1 cells as well as in both cell types in the co-culture system (representative of n=3). **E**, Examples of dragon tails display showing single PC3-GFP cell migration tracks in which temporal changes in cell location are indicated as coloured lines (last frames of the time-lapse movies). Each data point represents a single cell that has been analysed in the time-lapse movies. FST, follistatin; FAKi, focal adhesion kinase inhibitor.

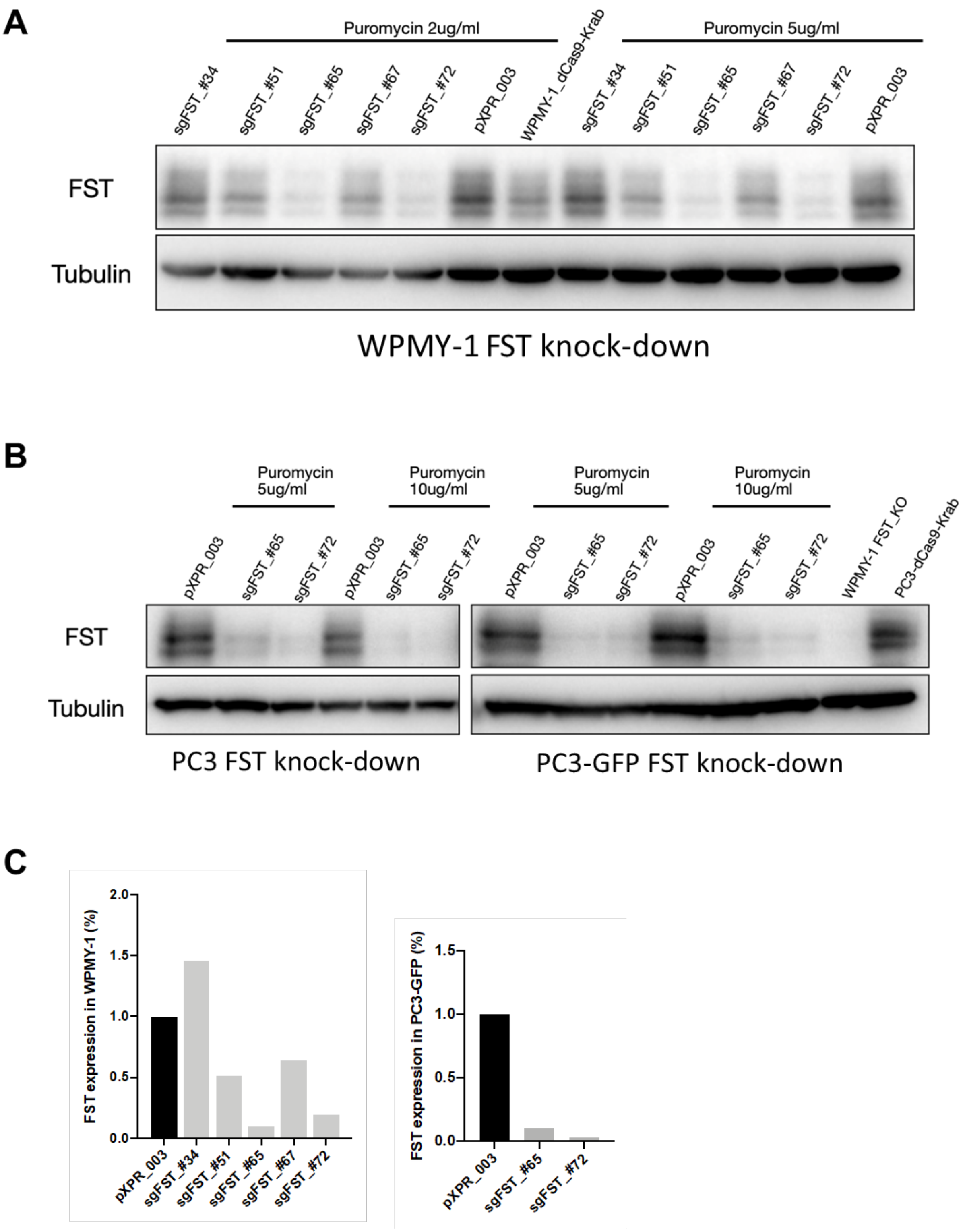
A
Puromycin 2ug/ml
Puromycin 5ug/ml
sgFST_#34
sgFST_#51
sgFST_#65
sgFST_#67
sgFST_#72
pXPR_003
WPMY-1_dCas9-Krab
sgFST_#34
sgFST_#51
sgFST_#65
sgFST_#67
sgFST_#72
pXPR_003
FST
Tubulin
WPMY-1 FST knock-down
B
Puromycin 5ug/ml
Puromycin 10ug/ml
Puromycin 5ug/ml
Puromycin 10ug/ml
pXPR_003
sgFST_#65
sgFST_#72
pXPR_003
sgFST_#65
sgFST_#72
pXPR_003
sgFST_#65
sgFST_#72
pXPR_003
sgFST_#65
sgFST_#72
WPMY-1 FST_KO
PC3-dCas9-Krab
FST
Tubulin
PC3 FST knock-down
PC3-GFP FST knock-down
C
FST expression in WPMY-1 (%)
2.0
1.5
1.0
0.5
0.0
pXPR_003
sgFST_#34
sgFST_#51
sgFST_#65
sgFST_#67
sgFST_#72
FST expression in PC3-GFP (%)
1.5
1.0
0.5
0.0
pXPR_003
sgFST_#65
sgFST_#72

Fig. S10. FST knockdown in WPMY-1 and PC3-GFP cells by CRISPRi. A and **B**, Western blot analysis of FST expression in WPMY-1 (**A**) and PC3-GFP (**B**) cells, transfected with empty vector (EV) (pXPR003) or with one of five different sgRNA sequences targeting FST (sgFST_#34, sgFST_#51, sgFST_#65, sgFST_#67 and sgFST_#72). PC3-dCas9-Krab and WPMY-1-dCas-Krab were used as control cell lines (n=1). **C**, Quantification of Western blots by densitometry (n=1). FST, follistatin.

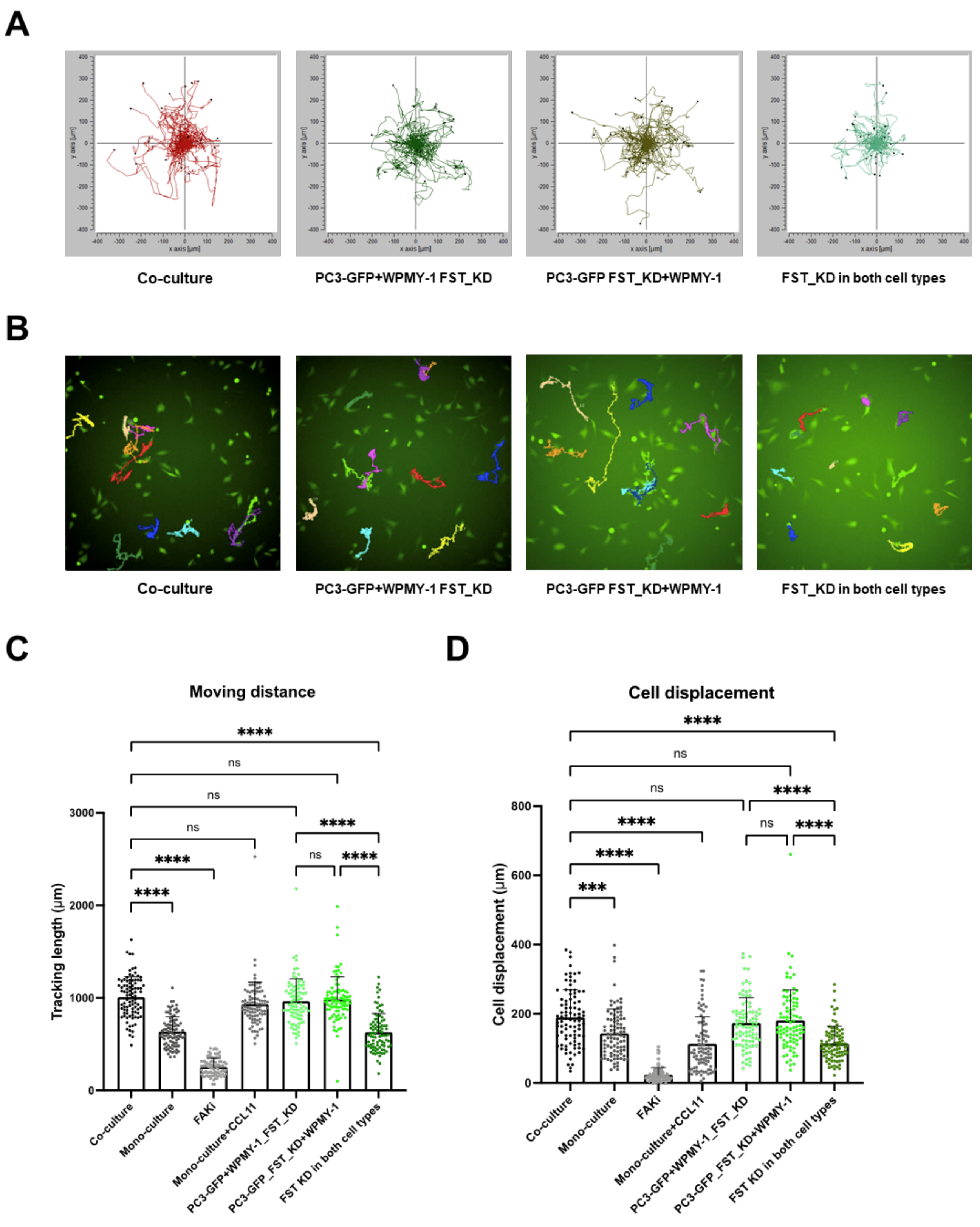
A
Co-culture
PC3-GFP+WPMY-1 FST_KD
PC3-GFP FST_KD+WPMY-1
FST_KD in both cell types
B
Co-culture
PC3-GFP+WPMY-1 FST_KD
PC3-GFP FST_KD+WPMY-1
FST_KD in both cell types
C
Moving distance
Tracking length (μm)
3000
2000
1000
0
****
ns
ns
ns
****
****
ns
****
****
****
Co-culture
Mono-culture
FAKi
Mono-culture+CCL11
PC3-GFP+WPMY-1_FST_KD
PC3-GFP_FST_KD+WPMY-1
FST KD in both cell types
D
Cell displacement
Cell displacement (μm)
800
600
400
200
0
****
ns
ns
****
****
ns
****
****
***
Co-culture
Mono-culture
FAKi
Mono-culture+CCL11
PC3-GFP+WPMY-1_FST_KD
PC3-GFP_FST_KD+WPMY-1
FST KD in both cell types

Fig. S11. Stable knockdown of FST in both cell types by CRISPRi impairs migration of prostate cancer cells in co-culture. **A**. Trajectories of PC3-GFP cells in the co-culture system when FST was knocked down individually in PC3-GFP or WPMY-1 cells as well as in both cell types (representative of n=3). **B**, Examples of dragon tails display showing single PC3-GFP cell migration tracks in which temporal changes in cell location were indicated as coloured lines (last frames of the time-lapse movies). **C** and **D**, Quantification of the accumulative migrated distance (**C**) and cell displacement (**D**) which PC3-GFP cells travelled when FST was knocked down individually in PC3-GFP or WPMY-1 cells as well as in both cell types in the co-culture system (n=3). Each data point represents a single cell that has been analysed in the time-lapse movies. Data are presented as mean ± SD; one-way ANOVA, Tukey multiple comparisons; ns, not significant, ***$p<0.001$, ****$p<0.0001$. KD, knockdown; FST, follistatin; FAKi, focal adhesion kinase inhibitor.

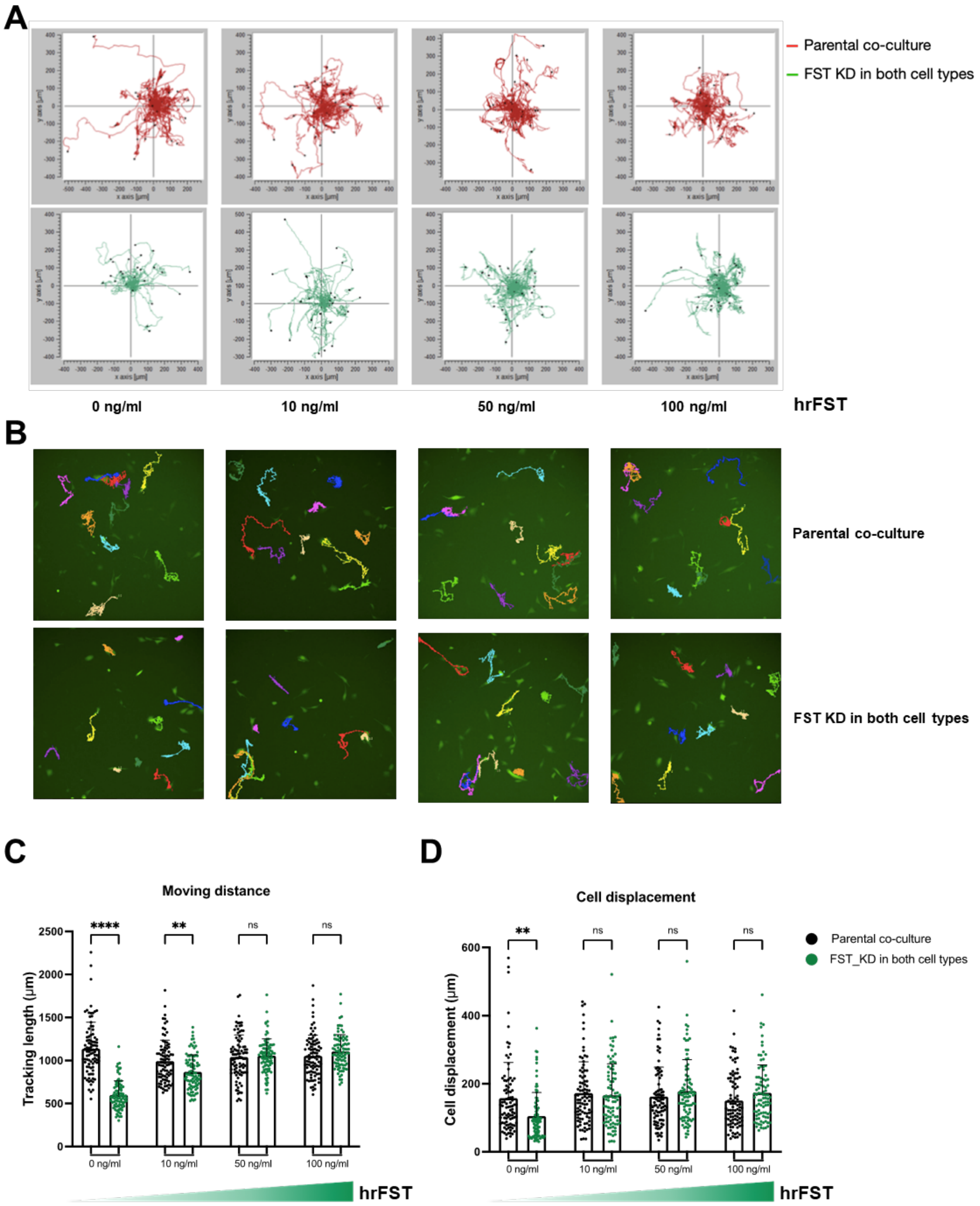
A
Parental co-culture
FST KD in both cell types
0 ng/ml
10 ng/ml
50 ng/ml
100 ng/ml
hrFST
B
Parental co-culture
FST KD in both cell types
C
Moving distance
Tracking length (µm)
2500
2000
1500
1000
500
0
****
**
ns
ns
0 ng/ml
10 ng/ml
50 ng/ml
100 ng/ml
hrFST
D
Cell displacement
Cell displacement (µm)
600
400
200
0
**
ns
ns
ns
0 ng/ml
10 ng/ml
50 ng/ml
100 ng/ml
hrFST
Parental co-culture
FST_KD in both cell types

Fig. S12. Human recombinant FST rescues the decreased migration of prostate cancer cells in co-culture caused by stable FST knockdown. **A**, Trajectories of PC3-GFP cells in parental co-culture and FST knockdown co-culture with or without additional human recombinant FST (hrFST). Three different concentrations (10ng/ml, 50ng/ml and 100ng/ml) of hrFST were added (representative of n=3). **B**, Examples of dragon tails display showing single PC3-GFP cell migration tracks in which temporal changes in cell location were indicated as coloured lines (last frames of the time-lapse movies). **C** and **D**, Quantification of the accumulative migrated distance (**C**) and cell displacement (**D**) which PC3-GFP cells travelled. Each data point represents a single cell that has been analysed in the time-lapse movies (n=3). Data are presented as mean ± SD; one-way ANOVA, Tukey multiple comparisons; ns, not significant, **p<0.01, ****p<0.0001. KD, knockdown; FST, follistatin; hrFST, human recombinant FST.

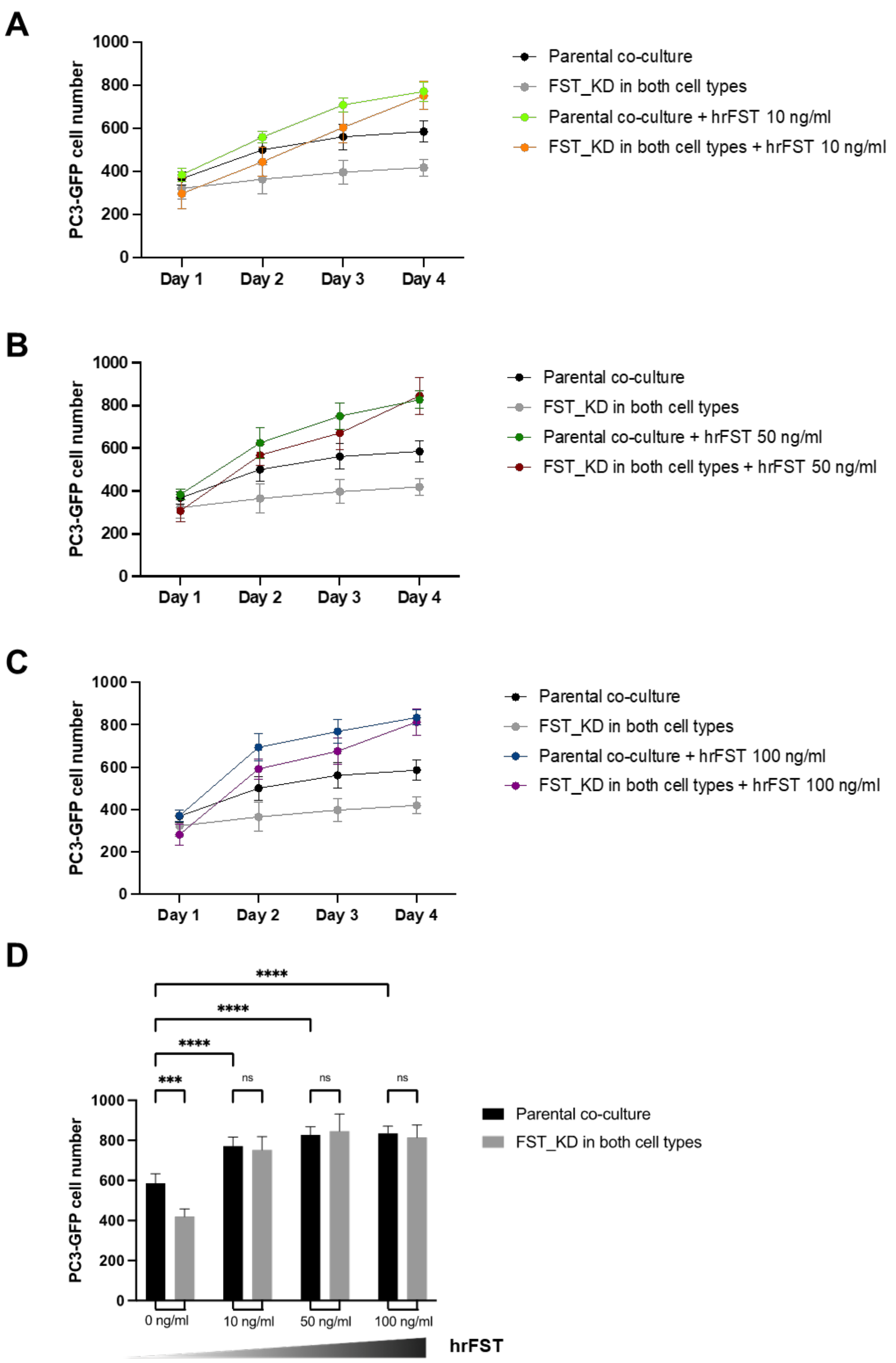
A
PC3-GFP cell number
1000
800
600
400
200
0
Day 1
Day 2
Day 3
Day 4
Parental co-culture
FST_KD in both cell types
Parental co-culture + hrFST 10 ng/ml
FST_KD in both cell types + hrFST 10 ng/ml
B
Parental co-culture + hrFST 50 ng/ml
FST_KD in both cell types + hrFST 50 ng/ml
C
Parental co-culture + hrFST 100 ng/ml
FST_KD in both cell types + hrFST 100 ng/ml
D
****
****
****
***
ns
ns
ns
0 ng/ml
10 ng/ml
50 ng/ml
100 ng/ml
hrFST

Fig. S13. Human recombinant FST rescues the impaired proliferation of prostate cancer cells in co-culture mediated by stable FST knockdown. **A-C**, Time course graphs of PC3-GFP cell growth with or without 10ng/ml (**A**), 50ng/ml (**B**) and 100ng/ml (**C**) of hrFST protein added to parental or knockdown co-cultures. **D**, Quantitation of PC3-GFP cell numbers 72 h after human recombinant FST protein treatment (n=3). Data are presented as mean ± SD; one-way ANOVA, Tukey multiple comparisons; ns, not significant, ***p<0.001, ****p<0.0001. KD, knockdown; FST, follistatin; hrFST, human recombinant FST.

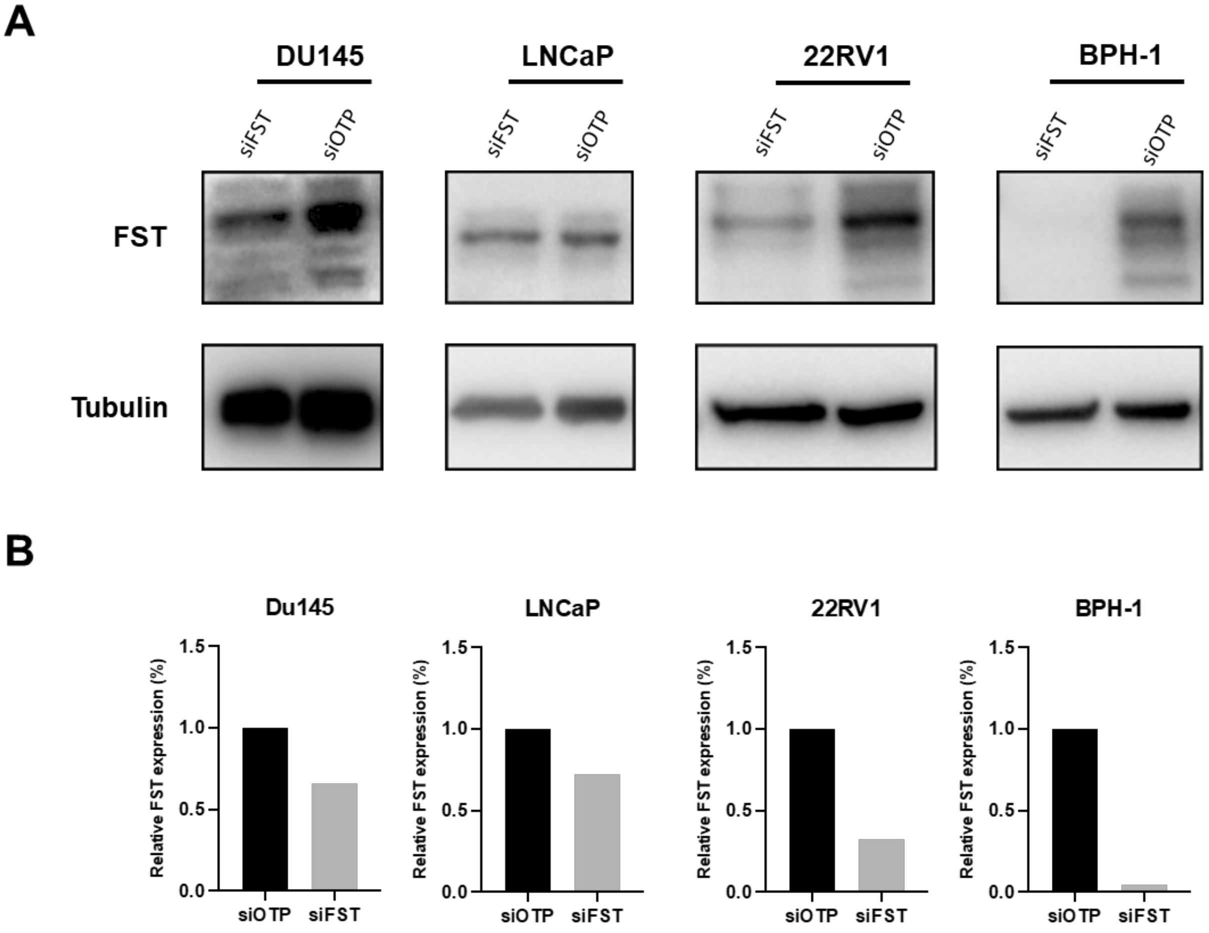

Fig. S14. FST expression and knockdown in different prostate epithelial/cancer cell lines. FST was knocked down by siRNA transfection with ON-TARGETplus (OTP) as the control. **A**, Western blots validated the FST knock down efficiency in prostate epithelial/cancer cell lines (n=1). **B**, Quantification of Western blots by densitometry (n=1). FST, follistatin.

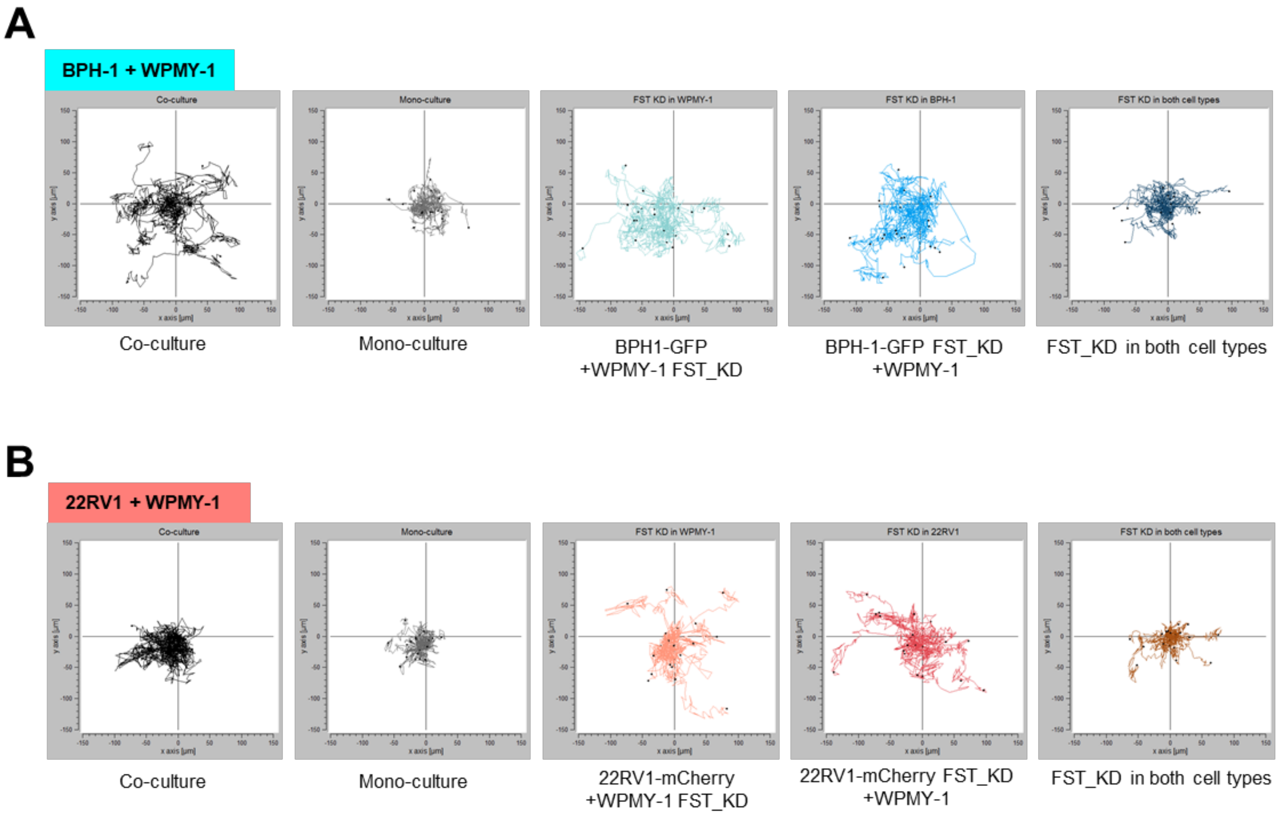

Fig. S15. FST regulates migration of additional prostate epithelial/cancer cell lines in co-culture with fibroblasts. **A** and **B,** Trajectories of BPH-1-GFP (**A**)/22RV1-mCherry (**B**) cells in the co-culture system when FST was knocked down in individual or both cell types (representative of n=3). KD, knockdown; FST, follistatin;

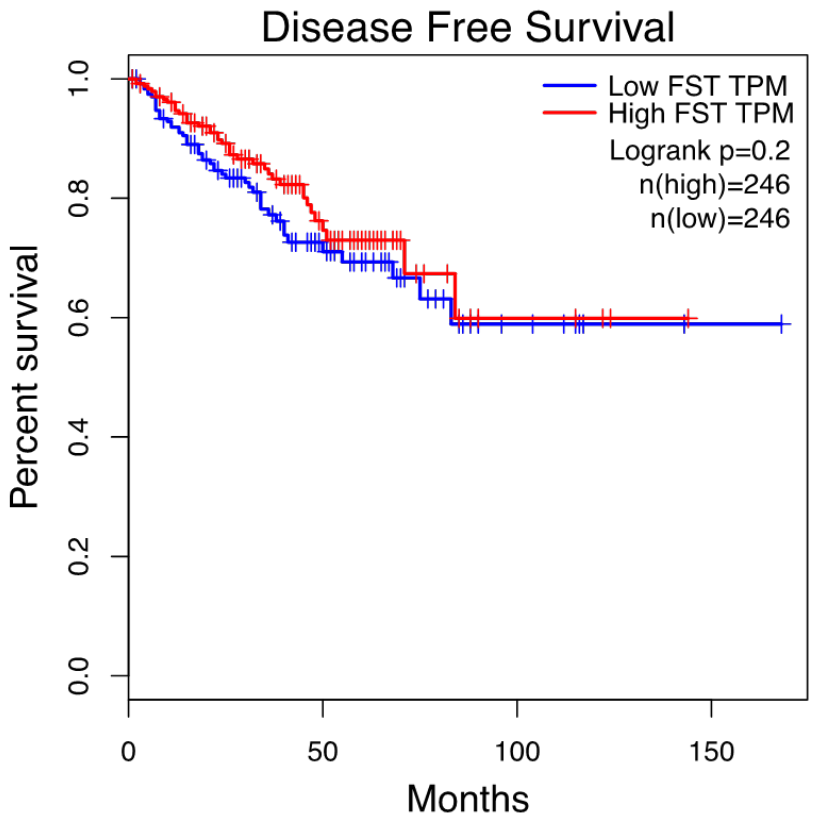

Fig. S16. Relationship of tumoural FST expression to disease-free survival of patients with prostate cancer. FST, follistatin; TPM, transcript per million.

Supplementary tables

Table S2. siRNA sequences used.

Targets	Sequences	
CXCL10	1. UGAAAGCAGUUAGCAAGGA	2. GUAAUCAACCUGUUAAUCC
	3. AAUCGAAGGCCAUCAAGAA	4. AAGUGGCAUUCAAGGAGUA
CXCL6	1. GAAGCAAGUUUGUCUGGAC	2. CAAAACGAUUGGUAAACUG
	3. GCACUUGUUUACGCGUUAC	4. GGCAUAAUGUCAUGAUUUA
CXCL16	1. GGAAGUUGUUAUUGUGGUA	2. CAGAUCUGCCGGUUCAUUA
	3. GAAUUGAUGAGCUGUCUUG	4. UCUAAUACCUGAGCCAAGA
FST	1. GGACUACAGCUUUCCUAUA	2. GUAAAGAAACGUGUGAGAA
	3. GGUAAACUCUCUAUAAGUG	4. UGUGUGACCUGUAAUCGGA
PDGFA	1. GGACGGUCAUUUACGAGAU	2. GCGGAUACCUCGCCCAUGU
	3. CCACUAAGCAUGUGCCCGA	4. CCACAAGCCUGAAUCCGGA
IL-17	1. AACCUGCUGUUUCUUCUUA	2. GACCUGGUGUCACGGAUGA
	3. GAGGUCAACUUGCAGCUGU	4. AGAGGAACAUCGAGGAGAU

Table S3. Summary of sgRNA sequences and primers.

		sgRNA Target sequence	Primer
FST g#72	F	GCGCGCGTCGCAGAGGCCAG	CACCGGCGCGCGTCGCAGAGGCCAG
	R	CTGGCCTCTGCGACGCGCGC	AAACCTGGCCTCTGCGACGCGCGCC
FST g#51	F	GGCGGAGGGCGCAGCGATCC	CACCGGGCGGAGGGCGCAGCGATCC
	R	GGATCGCTGCGCCCTCCGCC	AAACGGATCGCTGCGCCCTCCGCCC
FST g#65	F	TCGCAGAGGCCAGCGGCGGA	CACCGTCGCAGAGGCCAGCGGCGGA
	R	TCCGCCGCTGGCCTCTGCGA	AAACTCCGCCGCTGGCCTCTGCGAC
FST g#34	F	GGCGGCCGGCGGCGAGCGCG	CACCGGGCGGCCGGCGGCGAGCGCG

	R	CGCGCTCGCCGCCGGCCGCC	AAACGCGCGCTCGCCGCCGGCCGCCC
FST g#67	F	ATCGCTGCGCCCTCCGCCGC	CACCGATCGCTGCGCCCTCCGCCGC
	R	GCGGCGGAGGGCGCAGCGAT	AAACGCGGCGGAGGGCGCAGCGATC

F, forward sequence; R, reverse sequence.

Table S4. Real-time PCR primers.

Gene	Forward primer sequence (5'-3')	Reverse primer sequence (3'-5')
FST	1. TGCCACCTGAGAAAGGCTAC	TGGATATCTTCACAGGACTTTGC
	2. TCTGCCAGTTCATGGAGGA	TCCTTGCTCAGTTCGGTCTT
β-Actin	GGATGCAGAAGGAGATCACTG	CGATCCACACGGAGTACTTG
